# SOX2 downregulation of PML increases HCMV gene expression and growth of glioma cells

**DOI:** 10.1371/journal.ppat.1011316

**Published:** 2023-04-14

**Authors:** Le Wen, Xian-Zhang Wang, Yong Qiu, Yue-Peng Zhou, Qing-Yang Zhang, Shuang Cheng, Jin-Yan Sun, Xing-Jun Jiang, Simon Rayner, William J. Britt, Jian Chen, Fei Hu, Fang-Cheng Li, Min-Hua Luo, Han Cheng

**Affiliations:** 1 Joint Center of Translational Precision Medicine, Guangzhou Institute of Pediatrics, Guangzhou Women and Children’s Medical Center, Guangzhou, China; 2 State Key Laboratory of Virology, Wuhan Institute of Virology, Chinese Academy of Sciences, Wuhan, China; 3 University of Chinese Academy of Sciences, Beijing, China; 4 Wuhan Brain Hospital, Ministry of Transportation, Wuhan, China; 5 Department of Neurosurgery, Xiangya Hospital, Central South University, Changsha, China; 6 Department of Medical Genetics, Oslo University Hospital/University of Oslo, Oslo, Norway; 7 Department of Pediatrics, University of Alabama at Birmingham, Birmingham, Alabama, United States of America; 8 Chinese Institute for Brain Research (Beijing), Research Unit of Medical Neurobiology, Chinese Academy of Medical Sciences, Beijing, China; 9 Shanghai Public Health Clinical Center, Fudan University, Shanghai, China; University of Wisconsin-Madison, UNITED STATES

## Abstract

The presence of human cytomegalovirus (HCMV) in glioblastoma (GBM) and improved outcomes of GBM patients receiving therapies targeting the virus have implicated HCMV in GBM progression. However, a unifying mechanism that accounts for the contribution of HCMV to the malignant phenotype of GBM remains incompletely defined. Here we have identified SOX2, a marker of glioma stem cells (GSCs), as a key determinant of HCMV gene expression in gliomas. Our studies demonstrated that SOX2 downregulated promyelocytic leukemia (PML) and Sp100 and consequently facilitated viral gene expression by decreasing the amount of PML nuclear bodies in HCMV-infected glioma cells. Conversely, the expression of PML antagonized the effects of SOX2 on HCMV gene expression. Furthermore, this regulation of SOX2 on HCMV infection was demonstrated in a neurosphere assay of GSCs and in a murine xenograft model utilizing xenografts from patient-derived glioma tissue. In both cases, SOX2 overexpression facilitated the growth of neurospheres and xenografts implanted in immunodeficient mice. Lastly, the expression of SOX2 and HCMV immediate early 1 (IE1) protein could be correlated in tissues from glioma patients, and interestingly, elevated levels of SOX2 and IE1 were predictive of a worse clinical outcome. These studies argue that HCMV gene expression in gliomas is regulated by SOX2 through its regulation of PML expression and that targeting molecules in this SOX2-PML pathway could identify therapies for glioma treatment.

## Author summary

HCMV has been implicated in glioma pathogenesis, but the molecular characterization of the infection is completely lacking, hindering a thorough understanding of how HCMV contributes to glioma progression. We identified the SOX2-PML axis as a key regulator of HCMV infection in glioma cells and demonstrated in a patient-derived xenograft mouse model that the SOX2-PML axis not only controls HCMV infection but also contributes to the oncomodulatory role of the virus. Consistent with this finding, high levels of SOX2 and IE1 in gliomas predict a worse prognosis. To our knowledge, this is the first report of molecular determinants of HCMV infection in glioma cells, and the SOX2-IE1 pair may serve as a valuable prognostic factor to stratify glioma patients for targeted molecular therapies.

## Introduction

Glioblastoma (GBM) is a highly invasive high-grade glioma (HGG) with a 5-year overall relative survival of only 6.8% [1]. The development of GBM is a complex multistep process that is likely dependent on both genetic determinants of the host and external risk factors. Viral infection accounts for up to 15% of all human cancers and thus could be a potentially important external risk factor for the development and phenotypic behavior of GBMs [2]. Though human cytomegalovirus (HCMV) has not been recognized as an oncogenic virus, it has been associated with the malignant progression of GBM [3,4]. HCMV immediate early protein 1 and 2 (IE1/2) are detected in glioma cells but not in the peritumoral and adjacent normal-appearing brain regions and their expression levels correlate positively with glioma grades and negatively with survival [5,6]. In addition, HCMV seropositivity has been significantly associated with poorer survival of GBM patients [7]. Remarkably, antiviral and immune-based therapies targeting HCMV improved the outcomes of GBM patients [8–11]. These studies strongly support the potential role of HCMV as a contributor to GBM progression, although a unifying mechanism for the role of HCMV in the malignant phenotypes of GBM has not been established. Therefore, further characterization of the viral gene expression of HCMV in gliomas could further define its role in GBM pathogenesis and potentially provide novel therapeutic targets.

HCMV readily infects GBM sphere cultures in vitro, and the infected cells display properties of glioma stem cells (GSCs) [12]. GSCs are tumor-initiating cells resistant to chemo- and radiotherapy and provide a reservoir for tumor recurrence [13]. GSCs highly express markers associated with neural stem cells, such as CD133 [4] and sex-determining region Y-box 2 (SOX2) [14]. SOX2 is a transcription factor highly expressed during neural development but downregulated after cell differentiation [15]. SOX2 expression is normally highly restricted in the adult brain but induced in malignant gliomas [16]. Upregulation of SOX2 in chemotherapy-resistant GBM tissues and cells facilitates the malignant phenotype by regulating tumor-initiation and drug-resistant cell survival [17].

HCMV has a broad tropism and infects a wide range of cell types *in vivo*, but viral gene expression, and ultimately, genome replication and viral progeny assembly, are determined by virus-host interactions in a context-dependent manner [18]. The host factors that facilitate HCMV infection in glioma cells remain uncharacterized. The aberrantly high expression of SOX2 in GSCs prompted us to investigate whether SOX2 shapes HCMV infection in gliomas. In this study, we first show, to our knowledge, that SOX2 is crucial for HCMV infection in glioma cells. Subsequently, we have shown that SOX2 promotes HCMV gene expression by downregulating promyelocytic leukemia (PML) and Sp100 with disrupted formation of PML-nuclear bodies (PML-NBs). The impact of HCMV infection on glioma biology was then evaluated *in vitro* using a neurosphere assay with two patient-derived GSC cultures. HCMV gene expression regulated by the SOX2-PML axis enhanced GSCs’ self-renewal, and this phenotype could be inhibited by treatment with the antiviral agent, ganciclovir (GCV). Notably, the impact of the regulation of the SOX2-PML axis on HCMV infection and its oncomodulatory activities were then demonstrated in a patient-derived xenograft (PDX) mouse model. Lastly, a positive correlation between SOX2 and IE1 expression in glioma samples could be shown, thus providing clinical evidence that was in agreement with our findings in glioma cells and animal models.

## Results

### SOX2 promotes HCMV gene expression in glioma cells

To explore the role of SOX2 in HCMV infection in gliomas, we first determined whether high SOX2-expressing glioma cells are more permissive to HCMV infection than low SOX2-expressing cells by quantifying HCMV infection in six glioma cell lines with various SOX2 expression levels. SOX2 expression could be detected in greater than 70% of U251, LN229, and CCF cells, but less than 20% of U87, T98G, and A172 cells (S1A and S1B Fig). Upon HCMV infection, SOX2-positive cells were more susceptible than SOX2-negative cells, as indicated by viral IE1 expression (S1A and S1C Fig). These data demonstrated a correlation between SOX2 and IE1 expression in glioma cells, indicating that SOX2 expression could be a determinant of HCMV infection in gliomas.

**Fig 1:**
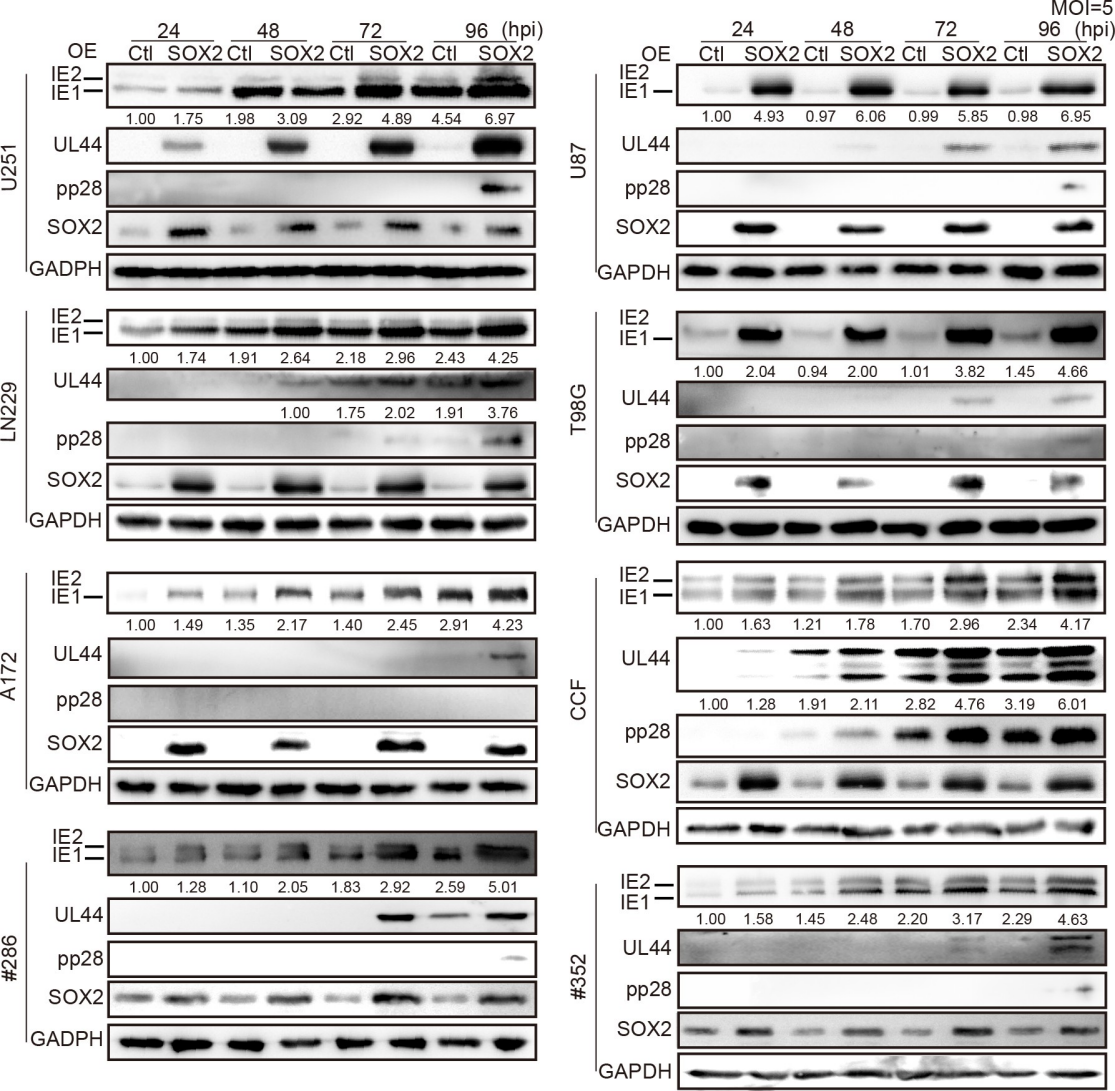
SOX2 promotes HCMV gene expression in glioma cells. Using lentivirus transduction, SOX2 overexpressing (OE) and control (Ctl) cells were made from glioma cell lines of U251, U87, LN229, T98G, A172, and CCF and primary glioma stem cells (GSCs) from two glioblastoma (GBM) cases, #286 and #352. The cells were infected with the HCMV Towne strain at an MOI of 5 and collected at the indicated times for immunoblotting (IB) analysis. Protein expression of SOX2 and HCMV protein IE1/2, UL44 (early protein), and pp28 (late protein) in SOX2-OE and Ctl cells are shown. GAPDH served as an internal control for protein quantification normalization. Relative levels of the indicated proteins are listed below the blots. Data are representative of three independent experiments.

Next, we studied the impact of SOX2 overexpression (OE) and knockout (KO) on HCMV infection. The generated OE and KO glioma cells were infected at a multiplicity of infection (MOI) of 5. HCMV genes are expressed in a temporal cascade, and representative genes of these three temporal classes—immediate early (*UL123* and *UL122* encoding IE1 and IE2), early (*UL44* encoding UL44), and late (*UL99* encoding pp28) were examined by RT-qPCR. Compared with the control cells (Ctl), SOX2-OE significantly upregulated *UL123* transcription at 4 h post-infection (hpi), *UL44* at 12 hpi, and *UL99* at 24 hpi (S2 Fig), and the corresponding viral protein levels in glioma cell lines and primary GSCs (#286 and #352) (Fig 1). CRISPR-Cas9 technology was utilized to generate SOX2-KO cells from glioma cell lines (U251, LN229, and CCF) and primary GSCs, which exhibit relatively higher SOX2 expression and possess higher HCMV infection efficiency than the other three cell lines. In these SOX2-KO cells, both viral gene transcription (S3 Fig) and protein levels (S4 Fig) were significantly less than in the negative control (NC) cells. We also evaluated the effects of SOX2-OE on viral entry and replication, and our data showed that although SOX2 facilitated HCMV gene expression and virus replication, it had no significant effect on viral entry (S5A and S5D Fig). Together, these data demonstrate that SOX2 positively increases both HCMV gene expression and replication in glioma cells.

### SOX2 modulates HCMV infection via inhibiting PML-NBs formation

Since SOX2 was reported to directly bind to the upstream regulatory region of human papillomavirus E6 to downregulate the expression of oncogenes E6 and E7 [19], we first speculated that SOX2 might act through a similar mechanism of binding to the major immediate early promoter (MIEP) of HCMV to increase HCMV gene expression. Upon HCMV infection of permissive cells, MIEP is initially transactivated by the virus itself or host transcription factors to drive the expression of the major IE genes, *UL122* and *UL123* [20]. In our initial experiments, we failed to demonstrate that SOX2-OE increased MIEP-driven transcription of a luciferase reporter in the human embryonic kidney (HEK) 293T and glioma cell lines (S6 Fig), suggesting that SOX2 may promote viral gene expression through other mechanisms.

**Fig 2:**
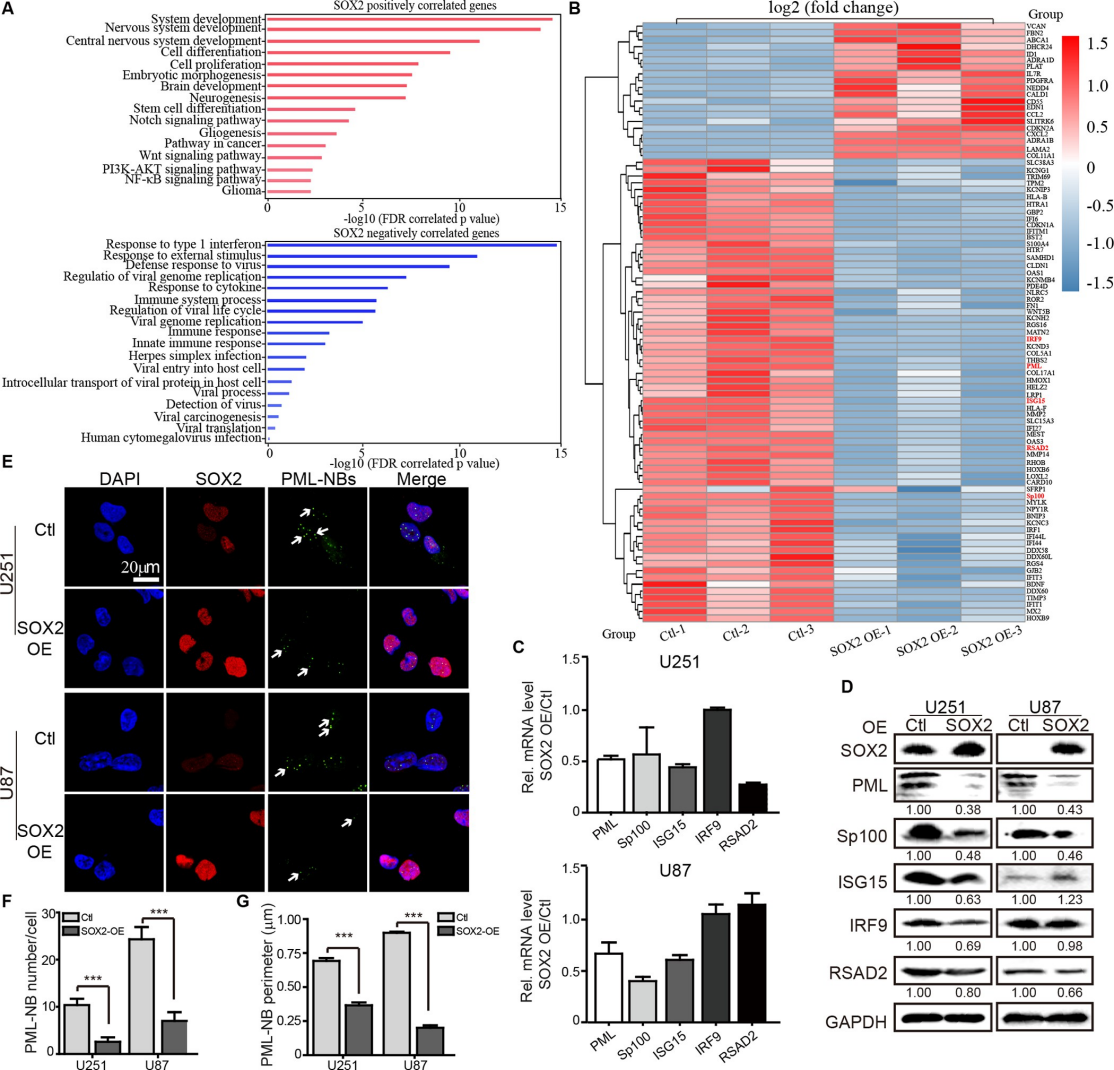
RNA-Seq analysis of SOX2-OE U251 cells. (**A**) Gene set enrichment analysis shows the top pathways positively or negatively correlated with SOX2-OE in U251 cells. (**B**) Heatmap of differentially expressed genes with function related to virus infection. The mRNA (**C**) and protein (**D**) levels of PML, Sp100, ISG15, IRF9, and RSAD2 in SOX2-OE and Ctl cells of U251 and U87. (**E**) PML-NBs. SOX2 (red) and PML (green) were detected by immunofluorescence analysis (IFA), and PML-NBs were indicated by white arrows. The number (**F**) and perimeter (**G**) of PML-NBs were quantified from 5 random fields in 50 cells. Data in (**C**, **F** and **G**) are from three independent experiments and represent as means ±SEM (Student’s t-test; *, p < 0.05; **, p < 0.01; ***, p < 0.001).

Given that SOX2 is a transcription factor, we wondered whether SOX2 acts indirectly via its downstream target genes in glioma cells. To identify genes regulated by SOX2, RNA sequencing (RNA-Seq) was performed in SOX2-OE and Ctl U251 cells. Gene Ontology (GO) functional enrichment and Kyoto Encyclopedia of Genes and Genomes (KEGG) functional analyses showed that the upregulated genes were mainly involved in system development, neurogenesis, and a few important signaling pathways and that the downregulated genes were mostly related to immune response and regulation of viral replication (Fig 2A). In the remainder of this study, we focused on the factors related to virus replication. As shown in Fig 2B, multiple key components of the host antiviral defense system were decreased in SOX2-OE cells compared with Ctl cells. Among these candidates, five host factors (PML, Sp100, ISG15, IRP9, and RSAD2) have been related to HCMV infection and were further investigated. Interestingly, only PML and Sp100 were significantly downregulated at mRNA (Fig 2C) and protein (Fig 2D) levels by SOX2-OE in U251 and U87 cells.

PML functions as a scaffold protein to recruit Sp100 and other host proteins for the assembly and maintenance of PML-NBs [21]. Thus, we determined whether SOX2-induced decrease of PML and Sp100 impairs PML-NBs formation. PML-NBs were visualized by immunofluorescence (Fig 2E). We found that PML-NB dots in SOX2-OE cells were much fewer in number (Fig 2F) and smaller in size than those in Ctl cells (Fig 2G), arguing that SOX2-induced downregulation of PML and Sp100 disrupts PML-NBs formation in glioma cells.

### SOX2 downregulates PML and Sp100 through different mechanisms

**Fig 3:**
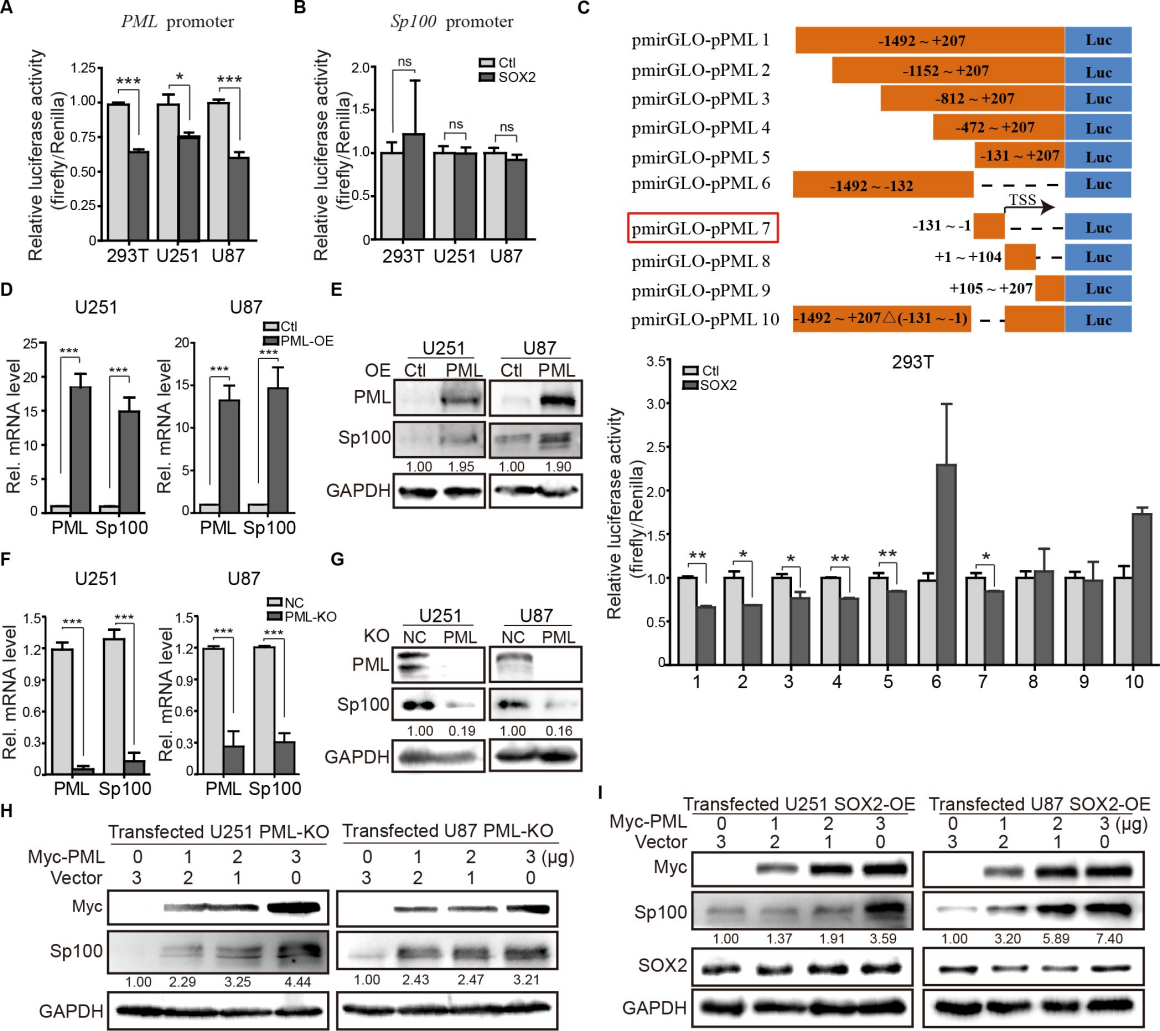
SOX2 downregulates PML and Sp100 by different mechanisms. Effects of SOX2 on the promoter activities of PML (**A**) and Sp100 (**B**) were assessed with a promoter-driven luciferase reporter system in 295T, U251, and U87 cells. (**C**) SOX2 suppresses PML expression by targeting the TSS region of the *PML* promoter. Luciferase expression driven by a series of truncated and deletion mutants of the *PML* promoter in 293T co-transfected with SOX2 or vector (Ctl) was quantified. Black lines indicate vector sequences, blue bars indicate luciferase reporter gene, and orange bars indicate the *PML* promoter fragments. Effects of PML overexpression on the mRNA (**D**) and protein (**E**) levels of PML and Sp100 in PML-OE and Ctl cells of U251 and U87. Effects of PML knockout on the mRNA (**F**) and protein (**G**) levels of PML and Sp100 in PML-KO and NC cells of U251 and U87. (**H**) Restored PML expression increases the Sp100 level. PML-KO U251 and U87 cells were transfected with the indicated amount of pHAGE-Myc-PML (Myc-PML) or vector (Ctl), and cells were collected 48h later for IB analysis of PML and Sp100. (**I**) PML expression restores endogenous Sp100 levels in SOX-OE cells. SOX2-OE U251 and U87 cells were transfected with the indicated amount of pHAGE-Myc-PML (Myc-PML) or pHAGE-GFP (vector), and cells were collected 48h later for IB analysis. Data in (**A**, B, **D** and **F**) are from three independent experiments and shown as mean ± SEM (Student’s t-test; ns, not significant; *, p < 0.05; **, p < 0.01; ***, p < 0.001).

SOX2 binds DNA in a sequence-specific manner to regulate gene transcription [22]. To test if SOX2 interferes with *PML* and *Sp100* transcription by interacting with their promoter regions, we constructed a promoter-driven luciferase reporter system. The luciferase expression is controlled by the promoter region of the *PML* gene (-1492 ~ +207) containing 1492bp upstream and 207bp downstream from the transcription start site (TSS) or the *Sp100* gene (-1210 ~ +146). This assay showed that SOX2-OE inhibited the promoter activity of *PML* but not *Sp100* in 293T and glioma cells (Fig 3A and 3B). We further analyzed the promotor repression by SOX2 with a series of truncated and deletion mutants of the *PML* promoter in 293T cells. Compared with the control, all promoter mutants containing -131~-1bp exhibited decreased luciferase activities in SOX2-OE cells, whereas two mutants (#6 and #10) lacking this region displayed enhanced activities (Fig 3C). This result suggests that the -131~-1bp region may act as a repressive element exploited by SOX2 for transcriptional suppression of PML expression.

Since Sp100 is recruited to PML-NBs by PML [23], we suspected PML might also regulate Sp100 gene expression. Thus, Sp100 expression was assessed by PML-OE and -KO and control cells (Ctl or NC) in U251 and U87 cells. As expected, Sp100 expression was dramatically increased in PML-OE cells (Fig 3D and 3E) and decreased in PML-KO cells (Fig 3F and 3G). Importantly, Sp100 expression was recovered in PML-KO U251 and U87 cells by ectopic expression of PML (Fig 3H). Finally, we tested if overexpressing PML could rescue Sp100 expression in SOX2-OE cells where PML and Sp100 expression is depressed. As anticipated, Sp100 protein levels were restored by ectopic expression of PML in SOX2-OE cells in a dose-dependent manner (Fig 3I). Taken together, these data indicate that SOX2 downregulates Sp100 in a PML-dependent manner.

### PML and Sp100 suppress HCMV gene expression in glioma cells

**Fig 4:**
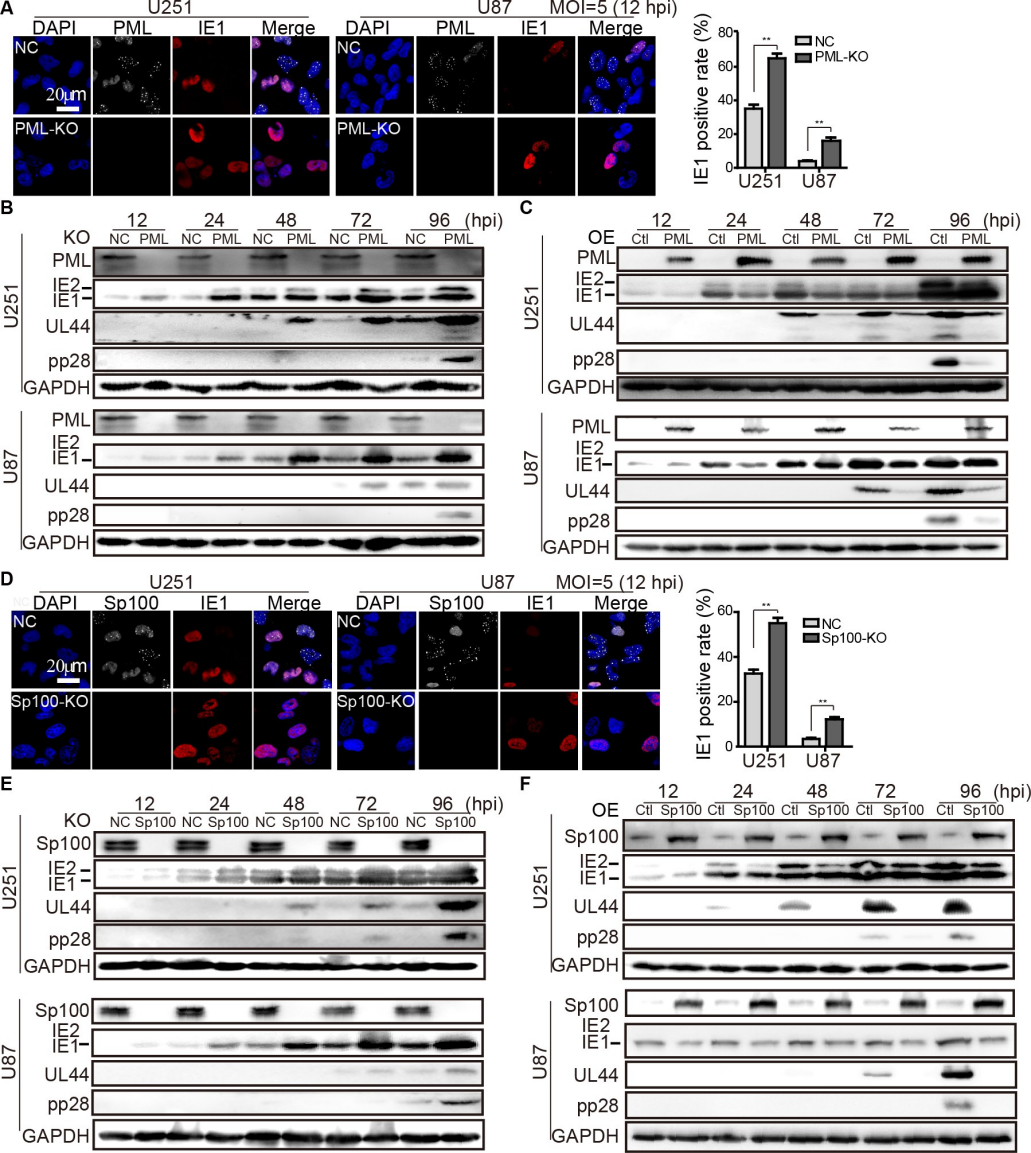
Both PML and Sp100 suppress HCMV gene expression in glioma cells. PML-KO, PML-OE, Sp100-KO, Sp100-OE, and their corresponding control cells were made with lentiviral transduction in U251 and U87 cells. These cells were infected with HCMV strain Towne at an MOI of 5 and harvested or fixed at the indicated times for viral gene expression determination. (**A**) Effect of PML-KO on HCMV IE1 expression by IFA. Representative images from three independent experiments are shown. Viral protein levels in PML-KO and NC (**B**), as well as PML-OE and Ctl cells (**C**), were examined by IB. (**D**) Effect of Sp100 on HCMV IE1 expression by IFA. IE1 positive cells in Sp100-KO and NC of U251 and U87 were quantified. Viral protein levels in Sp100-KO and NC (**E**), as well as Sp100-OE and Ctl cells (**F**), were examined by IB. GAPDH serves a loading control. For (**A**) and (**D**), IE1 positive cells were counted and normalized to the total cell number to calculate IE1 positive rates. Five random fields per coverslip from three coverslips were quantified in each independent experiment. Data collected from three independent experiments are shown as means ±SEM (Student’s t-test; ns, not significant; **, p < 0.01).

In cells permissive to HCMV infection, PML and Sp100 act as cellular restriction factors by inhibiting viral IE gene expression [24]. To confirm this antiviral activity in glioma cells, we examined the effects of PML-KO and OE in U251 and U87 cells infected with HCMV. The HCMV infection efficiency was quantified by counting IE1-positive cells at 12 hpi. PML-KO resulted in a two- to three-fold increase in HCMV IE1 gene expression (Fig 4A). We further quantified the mRNA levels of the viral genes (*UL123*, *UL44*, and *UL99*) from the three temporal classes in PML-KO, PML-OE, and control cells. The transcription of these viral genes was dramatically enhanced in PML-KO cells (S7A Fig) but suppressed in PML-OE cells (S7B Fig). Similarly, viral protein levels were significantly enhanced in PML-KO cells (Fig 4B) but decreased in PML-OE cells (Fig 4C).

The effect of Sp100 on HCMV infection was assessed similarly. The number of IE1-positive cells (Fig 4D) and viral protein levels significantly increased in Sp100-KO cells but markedly decreased in Sp100-OE cells compared with the control cells (Fig 4E and 4F). This inhibitory activity of Sp100 on the corresponding viral gene expression was also confirmed (S8A and S8B Fig). Overall, the antiviral activities of Sp100 and PML are comparable. These data make it clear that both PML and Sp100 suppress HCMV gene expression in glioma cells.

### HCMV infection regulated by the SOX2-PML axis accelerates glioma progression

**Fig 5:**
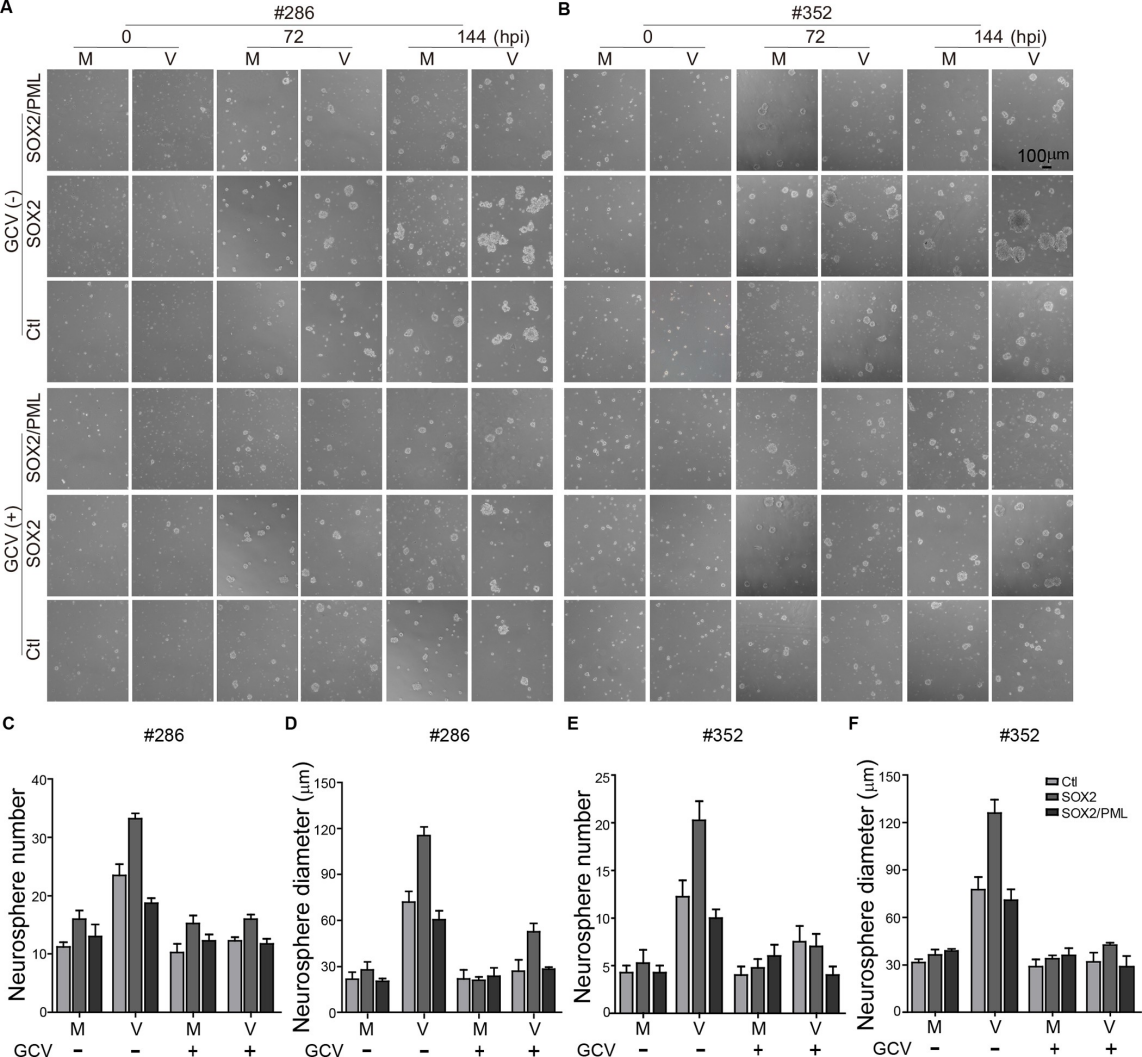
Effects of SOX2 overexpression and HCMV infection on neurosphere formation of GSCs. Patient-derived GSCs (#286 and #352) were transduced with SOX2 (SOX2), SOX2 and PML (SOX2/PML) or control lentivirus (Ctl). The transduced GSCs cultured in 48-well plates (2 x 10^5^/well) were mock-infected (M) or infected by HCMV Towne strain (V) at an MOI of 5, and a few wells were treated with 150 μM ganciclovir (GCV) for 48 hours. Representative images of spheres formed by GSC #286 (**A**) and GSC #352 (**B**) are shown. Quantification of neurospheres (number/field and size) at 144 hpi is shown in (**C**-**D**) for GSC #286 and (**E**-**F**) for GSC #352. Number and size of neurospheres were measured in random independent fields per well and three wells were included. Data are from three independent experiments and represent as means ±SEM. Two-way ANOVA analyses along with the Tukey post-hoc multiple comparisons were performed to evaluate the statistically significant differences between groups (**C**-**F**), and the p-values and statistical parameters are provided in Tables A-D in S1 Table.

Our findings suggested that SOX2 promotes HCMV gene expression and replication in gliomas by downregulating PML. We next evaluated the effect of this regulation on tumor growth *in vitro* and *in vivo*. Neurosphere formation of two patient-derived GSC cultures (#286 and #352) was assessed. SOX2 slightly enhanced neurosphere formation of #286 and #352. In contrast, HCMV infection greatly boosted the neurosphere formation capacities of both #286 and #352 GSC cultures (Fig 5A–5F). In addition, neurosphere formation capacities of both #286 and #352 GSC cultures were inhibited by treatment with the antiviral drug GCV, suggesting this potential tumor-promoting effect is caused by viral infection (Fig 5A–5F). Moreover, HCMV’s effects on GSCs could be further enhanced by SOX2 but offset by PML co-expression (SOX2/PML), strongly arguing for a regulatory effect of the SOX2-PML axis in HCMV’s oncomodulatory role (Fig 5A–5F).

**Fig 6:**
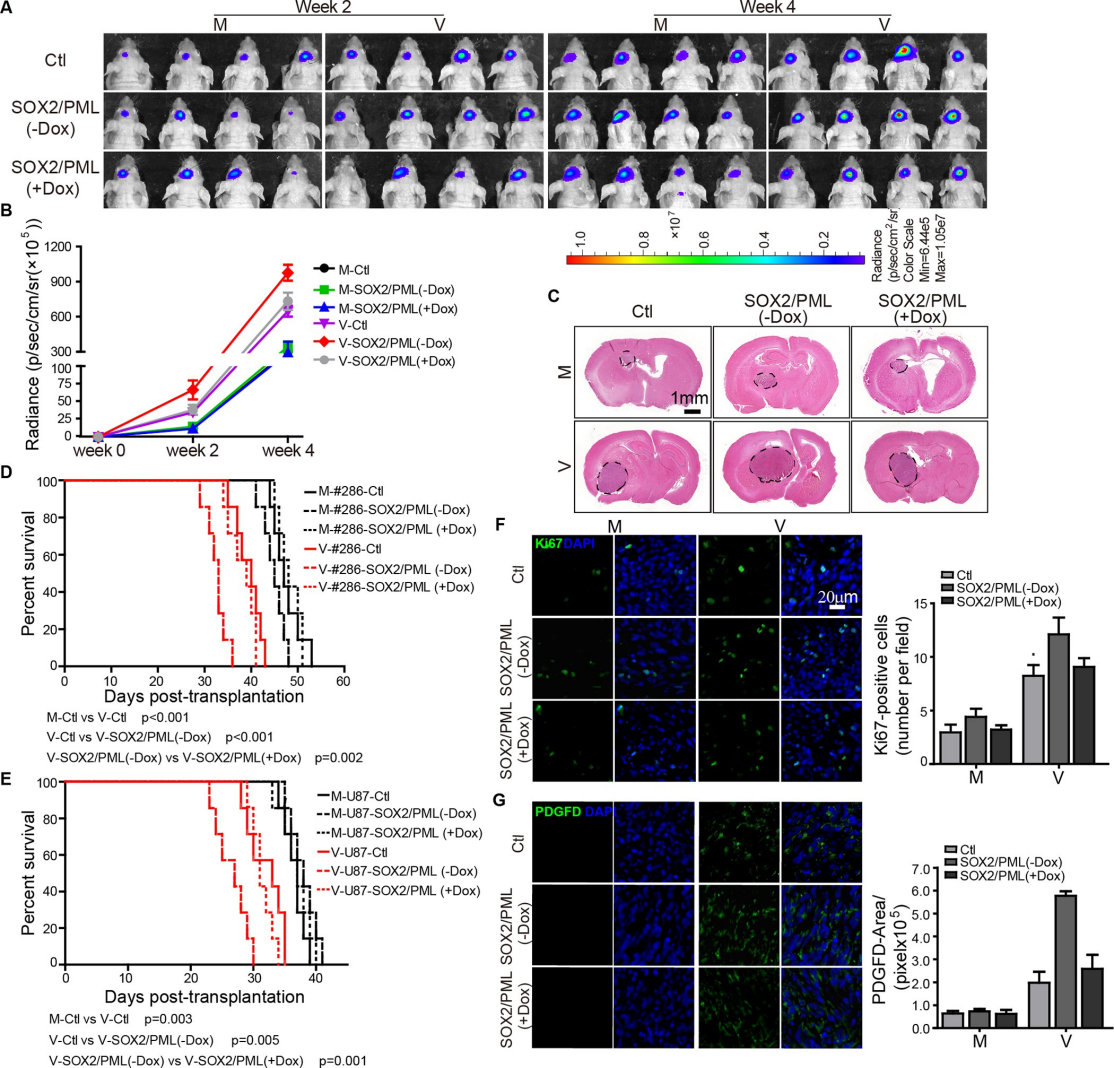
SOX2 enhances the oncomodulatory activities of HCMV in a patient-derived xenograft GBM model. Patient-derived #286-luc-Ctl (Ctl) and #286-luc-SOX2/PML (-Dox) cells were mock-infected (M) or infected by HCMV Towne strain (V) for three hours and then implanted into the brains of BALB/c nude mice. PML expression was induced by treating mice with doxycycline-containing water (+Dox, 2 mg/ml). The tumor growth (n = 7 for each group) was monitored at week 2 and week 4 (**A**) and quantified by luciferase activities (**B**). (**C**) Brain tissues were stained with H&E at four weeks after implantation. Survival curves of #286-luc (**D**) and U87-luc (**E**) tumor-bearing mice of different groups (n = 5 mice/group). Neurological sign free survival is defined as the time from tumor xenotransplantation until the end point without neurologic symptoms, such as unusual involuntary movements. The log-rank test was used to compare animal survival. Representative images and quantification of Ki-67 (**F**) and PDGFD (**G**) immunostaining (green) in the tumors at week 4 are shown. Data were collected from 5 images/mice and n = 3 mice/group. Data in (**B**, **F** and **G**) represent means ±SEM (Two-way ANOVA along with the Tukey post-hoc multiple comparisons, the p-values and statistical parameters are provided in Tables E-G in S1 Table).

Since CMV has strict species specificity, xenografting of human glioma cells into the brains of immunocompromised nude mice was used to establish an *in vivo* tumor model. Patient-derived GBM cells were first engineered to carry a luciferase reporter gene to generate #286-luc. Then, #286-luc was transduced with a lentivirus designed to stably express SOX2 but express PML in a doxycycline (Dox)-inducible manner. These cells were mock- or HCMV-infected for three hours and then implanted into the brains of nude mice. Tumors were collected on day 14 post-implantation to evaluate HCMV infection. As expected, SOX2-OE increased IE1 expression, which could be reverted to the control level when PML expression was induced by Dox (S9A Fig). HCMV infection markedly accelerated tumor growth of #286-luc xenograft (Fig 6A–6C) and reduced tumor-bearing mice survival compared with the mock-infected mice (Fig 6D). In addition, SOX2-OE enhanced the oncomodulatory activities of HCMV infection, which could be offset by induced PML expression (Fig 6A–6D). These observations were also confirmed in another xenograft mouse model with U87-luc cells (Figs 6E, S9B and S10A–S10C).

In a recent syngeneic GBM mouse model, elevated PDGFD expression induced by mouse cytomegalovirus (MCMV) infection results in pericyte recruitment, angiogenesis, and enhanced tumor growth [25]. HCMV-induced angiogenesis and cell proliferation were also observed in our xenogeneic GBM mouse models. In addition, HCMV-infected gliomas exhibited significantly increased PDGFD and Ki67 levels (Figs 6F, 6G, S10D and S10E) and intratumor vascularization (S11 Fig) than mock-infected controls. Though SOX2-OE had a negligible effect on cell proliferation and angiogenesis in mock-infected mice, it enhanced HCMV’s oncomodulatory activities, an effect which was attenuated by PML expression (Figs 6F, 6G, S10D, S10E and S11). These observations were consistent with tumor growth in this model. Together, these data strongly supported our hypothesis that SOX2 downregulated PML expression to facilitate HCMV infection and viral gene expression, consequently promoting glioma growth.

Considerable concerns exist for using the Towne strain because this lab-adapted strain has a defect pentamer complex and a substantial genetic deletion. To confirm the findings obtained with the Towne strain, we evaluated a more clinically relevant strain, TB40E. TB40E’s abilities to promote neurosphere formation *in vitro* (S12 Fig) and xenograft growth *in vivo* (S13 Fig) were enhanced by SOX2 overexpression and could be inhibited by GCV treatment. These data further support our claim that elevated SOX2 expression enhanced the oncomodulatory role of HCMV.

### High expression of IE1 and SOX2 predicts a poor prognosis

**Fig 7:**
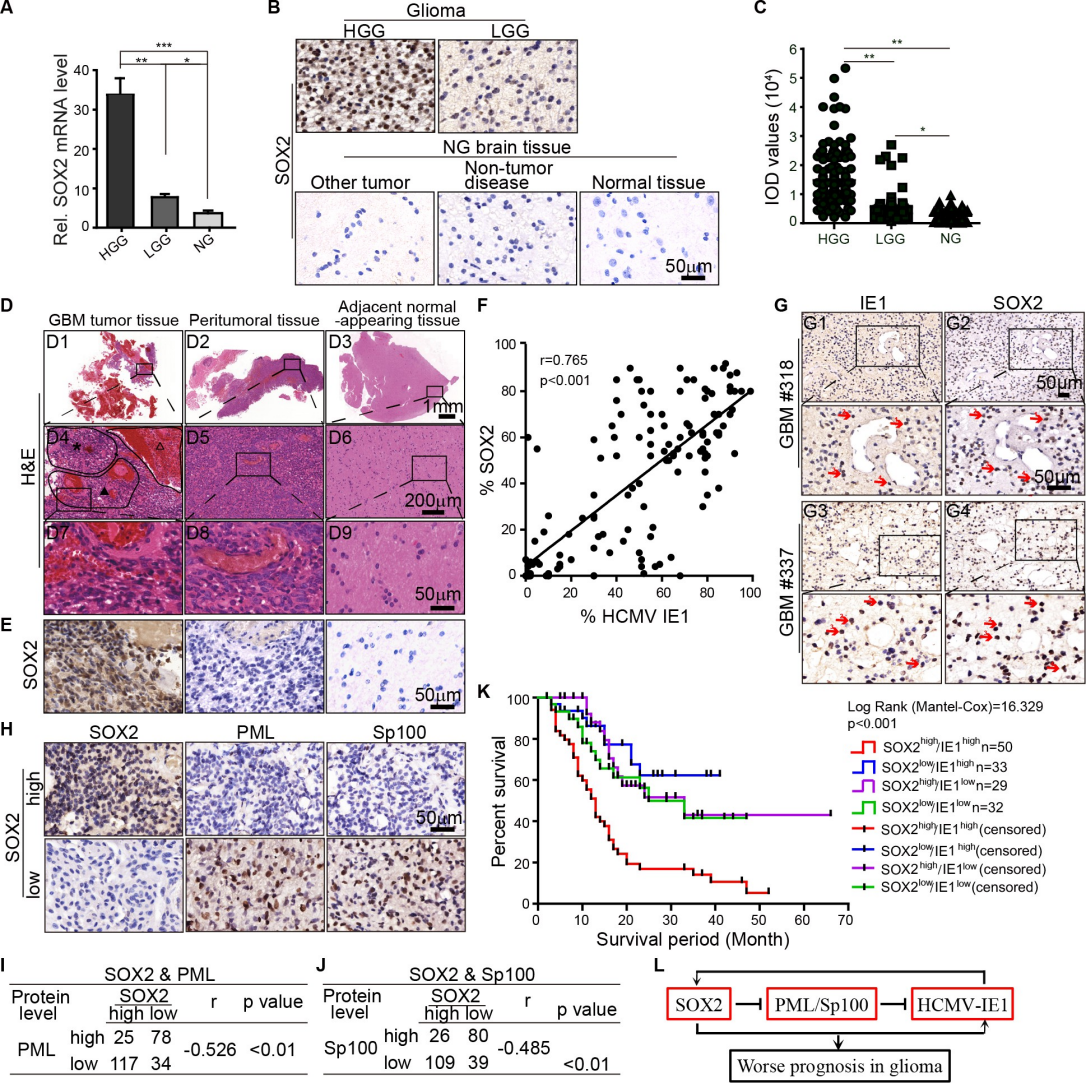
Positive correlation of SOX2 and IE1 expression in gliomas and survival analysis. (**A**) Relative mRNA levels of SOX2 in tumor tissue of HGG (n = 112), LGG (n = 60), and NG (n = 152). (**B**) Representative images of IHC staining of SOX2 in paraffin-embedded brain tissue of HGG, LGG, and NG. (**C**) SOX2 protein levels in brain tissues were quantified from IOD analyses of five random fields per specimen. LGG, n = 178. LGG, n = 76. LGG, n = 108. (**D**) Regions within a representative GBM brain specimen were classified as tumoral (D1), peritumoral (D2), and areas of the adjacent normal-appearing brain (D3) based on histopathology. In D1, the areas of abnormal cell proliferation (asterisk), necrosis (hollow triangle), and vascular proliferation or angiogenesis (solid triangle) are enlarged (D4). Further magnification in D4 shows vascular proliferation or angiogenesis (D7). For comparison, similar magnifications are shown of regions identified as peritumoral (D5, D8) or adjacent normal-appearing brain (D6, D9). (**E**) SOX2 staining in representative regions of D7-9. (**F**) Correlation between SOX2 and IE1 expression in 178 HGG subjects. SOX2 and IE1 immunostaining was quantified as previously mentioned, and the correlation was assessed by Pearson correlation analyses. (**G**) Co-expression of SOX2 and IE1 in glioma tissues. SOX2 and IE1 were stained in adjacent sections in GBM tissues by IHC; representative images and the corresponding magnification from two cases (#318 and #337) are shown. (**H**) Expression of SOX2, PML, and Sp100 in gliomas. Tumor tissues from 254 glioma patients were stained by using SOX2, PML, and Sp100 antibodies, respectively. Correlation analyses show SOX2 significantly inversely correlates with PML (**I**) and Sp100 (**J**). (**K**) Survival analysis. Patients (n = 144) were stratified into four groups based on SOX2 and IE1 expression levels in tumors (based on IHC scoring) and analyzed by Kaplan-Meier curves. Statistical significance was determined using the Log-rank (Mantel-Cox) test. (**L**) Model of the regulation of SOX2-PML axis on HCMV infection in gliomas. Data in (**A** and **C**) represent as means ±SD (One-way ANOVA along with the Tukey post-hoc multiple comparisons; *, p < 0.05; **, p < 0.01; ***, p < 0.001).

Since IE1 expression in gliomas has previously been positively correlated with tumor grades [6], we reasoned that if SOX2 determines HCMV infection in gliomas, the SOX2 expression pattern in gliomas should mirror that of IE1. Thus, we examined SOX2 mRNA and protein levels in the brain tissues of patients with gliomas. As expected, the SOX2 mRNA levels were much higher in HGG samples than in low-grade glioma (LGG) and non-glioma (NG) samples (Fig 7A). The SOX2 protein also exhibited a similar pattern (Fig 7B), and quantification analysis confirmed higher SOX2 levels in HGG samples than in LGG and NG samples (Fig 7C).

Characterization of SOX2 expression in surgically resected brain tissues from GBM patients was next accomplished in the tumor, peritumoral, and adjacent normal-appearing regions that were defined by histopathology (Fig 7D1–7D3). Abnormal cell proliferation, necrosis and hemorrhage, vascular proliferation, and angiogenesis were ubiquitously observed in the tumor region (Fig 7D1, 7D4 and 7D7) but progressively less appreciated in some cases or not seen in the peritumoral and adjacent normal-appearing brain areas (Fig 7D2, 7D3, 7D5, 7D6, 7D8 and 7D9). Similarly, SOX2-positive cells were predominantly located in regions containing the tumor, and both the prevalence of SOX2-expressing cells and their SOX2 expression levels declined dramatically or were undetectable in the outer areas (Fig 7E). This expression pattern of SOX2 mirrored that of IE1 in gliomas [5,6] (also corroborated by pp65 staining in S14 Fig), and linear regression analysis showed a strong positive correlation between IE1 and SOX2 expression in a cohort of 178 HGG patients (Fig 7F). Thus, we investigated whether they are co-expressed in the same cells. For each paraffin-embedded tissue specimen, 3 μm-thick adjacent slices were stained with anti-IE1 and anti-SOX2 antibodies, respectively. Representative images from two cases (#318 and #337) are shown (Fig 7G). Cells positive for IE1 (Fig 7G1, numbered arrows) were also positive for SOX2 (Fig 7G2, numbered arrows) in #318 and #337 GBM samples (Fig 7G3 and 7G4). Together these studies demonstrated that SOX2 downregulates PML and Sp100 in gliomas. To evaluate this regulation in samples from glioma patients, we examined their expression in tumor tissue from 178 HGG and 76 LGG patients. Of note, the expression of PML (Fig 7H and 7I) and Sp100 (Fig 7H and 7J) correlated inversely with SOX2 expression.

In our GBM mouse models, elevated SOX2 expression enhances HCMV’s oncomodulatory effects, suggesting that combinatorial expression profiling of IE1 and SOX2 may have prognostic value in patients with gliomas. Therefore, we evaluated the association between IE1 and/or SOX2 expression and patient survival. Tumors from a cohort of 144 glioma subjects were categorized according to the IE1 and SOX2 levels and assigned to four groups: IE1^low^/SOX2^low^, IE1^low^/SOX2^high^, IE1^high^/SOX2^low^, IE1^high^/SOX2^high^ (S2 Table). Kaplan-Meier analyses confirmed a significant association between IE1 or SOX2 level with prognosis (S3 Table). In addition, significant differences between IE1^high^/SOX2^high^ and the other three groups were shown (S4 Table), indicating that patients with high levels of both IE1 and SOX2 have significantly shorter survival time compared with the other groups (Fig 7K). Together, these data demonstrate that high levels of both SOX2 and IE1 in gliomas are associated with a poor prognosis for patients with glioma.

## Discussion

Since Cobbs and colleagues reported the high prevalence of HCMV in GBM in 2002 [5], there has been considerable debate about the presence and roles of HCMV in gliomas. A recent systemic review of almost all relevant studies reveals that technical issues may explain the inconsistency of HCMV detection in gliomas. Polymerase-based assays, including PCR and next-generation sequencing, consistently failed to detect HCMV nucleic acids, whereas in situ hybridization methods detected HCMV nucleic acids as good as optimized immunohistochemical staining protocols that detect viral proteins in GBM samples [26]. In addition, the fragile status of herpesviral genomes inside cells makes them refractory to PCR amplification, and an optimized sample preparation protocol to avoid physical deterioration of viral DNA is crucial for HCMV detection in gliomas [27]. Despite the controversy, the presence of HCMV in gliomas has also encouraged the development of several anti-CMV therapies and achieved promising results in GBM patients. Newly-diagnosed GBM patients receiving the anti-CMV valganciclovir had a significantly higher 2-year survival rate than controls (49.8% vs. 17.3%) [28]. Other anti-CMV strategies using vaccines or T-cell-based therapies have also demonstrated clinical benefits in GBM patients [8,11,29]. These studies demonstrate that HCMV infection is highly relevant to GBM pathogenesis and could be targeted for novel therapeutic development.

HCMV has broad cell tropism and is a significant infectious cause of several neurological disorders in immunocompromised patients and newborn infants infected *in-utero* [30]. In the brain, neural stem or progenitor cells (NSCs/NPCs) in the subventricular zone have been speculated to be a reservoir of CMV in the CNS [31], and it has been suggested recently that GBM arises from these NSCs/NPCs [32]. Though NPCs and neuronal and glial cells derived from NPCs are fully permissive to HCMV infection [33], viral transcription is restricted at several points in NSCs [34]. Cells derived from gliomas are non- or semi-permissive and phenotypically similar to NSCs in that only a subset of IE and early viral genes are expressed following HCMV infection of these cells [35]. In addition, glioma cell line T98G supports persistent infection and reactivation of HCMV [36]. In the current study, we demonstrate that elevated SOX2 expression in glioma cells promotes the expression of HCMV proteins, some of which may participate in multiple facets of glioma oncogenesis. Previous studies proposed several mechanisms including: 1) HCMV IE1 induction of damage DNA [37] and promotion of the stemness properties of GBM cells [38]; 2) HCMV US28 acceleration of GBM growth by facilitating development of an invasive, angiogenic phenotype [39]; and, 3) HCMV encoded viral IL-10 induction of relative immunosuppression in the tumor microenvironment (TME) facilitating gliomagenesis [40].

SOX2 interacts with the promoters of various genes to regulate essential molecular pathways in embryonic development [41]. Our data showed that genes downregulated by SOX2 in U251 are related to cellular restriction factors that limit HCMV gene expression as described in a previous report [14]. Among these candidates, the downregulation of two key constituents of PML-NBs (*i*.*e*., PML and Sp100) by SOX2 could be demonstrated. Our findings indicated that SOX2 only represses the promoter activity of *PML*, and the decreased Sp100 level is likely secondary to the SOX2-mediated decrease in PML expression. Several components of PML-NBs have been shown to repress HCMV productive infection by restricting IE gene expression in fully-permissive cells [42]. Similar suppression of viral gene expression was observed in glioma cells with elevated PML or Sp100 levels in this study. Reciprocally, the antiviral function of PML-NBs can be antagonized by viral proteins. In the early stage of infection, HCMV IE1 inhibits PML de novo SUMOylation [43] and degrades Sp100 [44,45] to initiate viral transcription. However, we did not observe significant changes in PML and Sp100 expression in HCMV-infected glioma cells (S15 Fig). Due to the SOX2-mediated downregulation of PML and Sp100, the PML-NBs formation is greatly reduced in glioma cells, removing the restriction on HCMV gene expression. Importantly, this phenotype can be rescued by overexpressing PML, confirming the direct participation of the SOX2-PML axis in regulating HCMV infection.

We evaluated the oncomodulatory activities of HCMV infection in GSC and PDX models. HCMV enhanced the self-renewal ability of GSCs, which could be inhibited by treatment with the antiviral GCV. Though SOX2-OE alone only slightly affected GSCs proliferation, it markedly enhanced HCMV’s effect. Again, this impact of SOX2-OE on HCMV gene expression could be limited by increasing the expression of PML. In a recent description of a GBM mouse model, MCMV promoted GBM growth in a syngeneic host by inducing PDGFD expression, an essential mediator of pericyte recruitment and angiogenesis [25]. In our xenograft GBM model using immunodeficient mice, the GBM xenograft is transferred into mice that lack a functional immune system to control tumor growth from GSCs of human origin and HCMV cannot infect cells of mouse origin. Notably, a similar promotion of the GBM following HCMV infection of the xenograft was observed in this model as described in the syngenic murine GBM model [25]. HCMV infection increased angiogenesis and cell proliferation, presumably leading to enhanced tumor growth and reduced survival of GBM-bearing mice. Furthermore, the concordant findings in these GBM *in vivo* models argue that the xenograft GBM model described in this study is a valid *in vivo* model to investigate the role of HCMV in GBM development. Thus, our findings that SOX2-OE enhances the oncomodulatory phenotypes of HCMV, a phenotype that can be reversed by increasing PML expression strongly argue for a role for the SOX2-PML axis in the regulation of HCMV infection *in vivo* and potentially, a mechanism that can account for the contribution of HCMV to the malignant phenotype of GBMs.

Our analysis of tumor tissue in patients with gliomas shows that SOX2 expression level can be positively correlated with HCMV IE1 levels and the grade of glioma. Furthermore, in some tumors, SOX2 and IE1 can be shown to be expressed within the same glioma cells. In addition, downregulation of PML and Sp100 was observed in glioma samples. Together, these data provide clinical findings in support of our hypothesis that the SOX2-PML axis regulates HCMV gene expression in gliomas. Lastly, HCMV IE1 has been shown to upregulate SOX2 by modulating the miR-145–SOX2 axis [38], a finding that contrasts with our previous findings in NPCs that IE1 decreases SOX2 levels [46]. This discrepancy between these findings may result from distinct transcriptomes in these two cell types. Though GSCs inherit characteristics similar to those of normal NSCs where interferon-stimulated genes (ISGs) are highly expressed [47], a high SOX2 level suppresses the expression of ISGs in GSCs. We speculate that high SOX2 expression and low ISGs expression are utilized by cancer cells to ensure survival [48]. Considering the intricate interplay between SOX2 and IE1 proteins, we prefer a scenario where an elevated SOX2 level leads to PML/Sp100 downregulation to allow HCMV gene expression, and through a paracrine loop, the expression of IE1 proteins reciprocally enhances SOX2 expression (Fig 7L). This positive feedback loop may lead to a potentially synergetic tumor-promoting effect from SOX2 and HCMV infection. New strategies targeting the paracrine loop of SOX2-IE1 could provide novel anti-glioma therapies.

## Materials and methods

### Ethics statement

All human subjects in the research were approved by the Institutional Review Board of Wuhan Institute of Virology and the Hospital Ethics Committee of Wuhan Brain Hospital (L20160009). All tissue donors included in this study were informed in detail with regard to study aims and design and signed informed consent forms. All animal studies were approved by the Ethics Committee on Animal Experiments at Wuhan Institute of Virology (WIVA10202104) and conducted following the Care and Use of Laboratory Animals guidelines. All surgeries were performed under general anesthesia, and best efforts were made to minimize the number of animals used and their suffering.

### Patients and samples

There were 278 glioma patients with malignancy grades ranging from grade I to grade IV (including astrocytoma, oligodendroglioma, ependymoma, and GBM). Control subjects included subjects with non-glioma brain tumors, non-tumor brain diseases, or normal brain tissues (S5 Table). All brain tumors were diagnosed histologically and verified according to the WHO Classification of Tumors of the Central Nervous System (2007) by two independent neuropathologists who were blind to the clinical data [6].

### Histological staining and evaluation

Brain tissue samples were fixed with 4% paraformaldehyde, embedded in paraffin, and sliced into 3–6 μm sections. SOX2, PML, Sp100, HCMV proteins IE1 and pp65 were detected by immunohistochemistry (IHC) staining following a standard protocol described previously [5]. H&E staining was performed as previously described [49]. Images were obtained using a Chirascan-SF digital slide scanning system (Thermo Fischer Scientific, MA, USA). To determine the protein expression levels, images from five random fields of each section were independently and blindly evaluated by two investigators. Yellow- or brown-stained areas in nuclei or cytoplasm were considered positive signals, and the integral optical density (IOD) of the staining was measured in all five random fields per section using Image-Pro Plus 6.0 (Media Cybernetics Inc., MD, USA) through batch scanning. The antibodies used in the experiment are listed in S6 Table.

### Cell culture

Primary glioma cells were isolated from fresh tumor tissues as described previously with slight modification [50–52]. Briefly, the tumor tissues were resected during surgery, maintained on wet ice in HBSS (Life Technology, CA, USA), and delivered to the laboratory within 5 hours. After rinsing with HBSS, the tissues were minced into small pieces, digested using collagenase (Sigma, MO, USA) and DNase I (Roche, Basel, Switzerland) at 37°C for 30 min, ground gently, and filtered through 70 μm nylon filters. Red blood cells were removed with RBC Lysis Buffer (Biolegend, USA) and the remaining cells were divided into two aliquots and pelleted by centrifugation. An aliquot was suspended in DMEM/F12 supplemented with 10% fetal bovine serum (FBS) (Life Technologies), 1% penicillin-streptomycin (100U/ml and 100μg/ml), and 4 mM L-glutamine for monolayer culture [6]. Another aliquot of cells was suspended in DMEM/F12 supplemented with 2mM GlutaMax, penicillin-streptomycin (100U/ml and 100mg/ml), 50mg/ml gentamycin, 1.5mg/ml amphotericin B, 2% B27 Supplement (all from Life Technologies), 10% BIT9500 (Stem Cell Technologies, Vancouver, CA), 20ng/ml epidermal growth factor, and 20ng/ml basic fibroblast growth factor (Prospec, MO, USA) for sphere culture.

Human embryonic lung fibroblasts (HELs) were isolated and maintained in the lab as described previously [53]. HELs, U-87MG (U87) (ATCC HTB-14) and T98G (ATCC CRL1690) cells were cultured in MEM; U-251MG (U251), LN229 (ATCC CRL-2611), and A172 (ATCC CRL1620) cells were cultured in DMEM; CCF-STTG1 (CCF) (ATCC CRL1718) cells were cultured in RPMI-1640 supplemented with 10% fetal bovine serum and 10% penicillin-streptomycin (Life Technologies).

### CRISPR-Cas9 knockout

The CRISPR design tool from the Broad Institute was used to design the guide RNA (gRNA). Double-stranded oligonucleotides corresponding to the target sequences were cloned into lenti-CRISPR-V2 vector plasmid to generate plasmids SOX2-KO, PML-KO, and Sp100-KO. Human SOX2, PML, and Sp100 gRNA targeting sequences are listed in S7 Table. These plasmids or lenti-CRISPR-V2 (NC) were transfected into 293T cells, along with helper plasmids pSPAX and pMD2G to produce lentiviruses. The viruses were harvested 48 h after transfection, ultra-filtrated (0.22 mm filter) (Millipore, MA, USA), and used to transduce glioma cell lines. Single-cell clones were selected by puromycin and expanded *in vitro*. Primary glioma cells were transduced with the knockouts lentiviruses as described previously [54], and the expression of the transgene(s) was determined by IB.

### Plasmid constructs and transfection

Mammalian expression plasmids for Myc-tagged SOX2, PML, and Sp100 were constructed with standard molecular biology techniques. The coding sequences of human SOX2, PML, Sp100 and SOX2-T2A-PML were cloned into pHAGE and all constructs were confirmed by sequencing. Plasmid pHAGE-SOX2, pHAGE-PML, pHAGE-Sp100, pHAGE -SOX2-T2A-PML, or pHAGE (Ctl) was transfected into 293T cells along with helper plasmids pSPAX and pMD2G to produce the corresponding lentiviruses. These lentiviruses (express GFP as the selection marker) were harvested at 72 h post transduction (hpt), titrated in 293T cells by quantifying GFP-positive cells, and stored as aliquots at -80°C for further application. Glioma cell lines and primary GSCs were transduced with the corresponding lentiviruses at an MOI of 1. Cultures with >90% GFP-positive cells at 5 dpt were harvested, and the expression of the transgene(s) was determined by IB. Plasmids were transiently transfected into 293T or glioma cells using Lipofectamine 2000 reagents (Invitrogen, CA, USA) or X-tremeGENE HP DNA Transfection Reagent (Roche) following the manufacturer’s instructions. For *in vivo* experiments, the firefly luciferase gene was cloned into pHAGE, and the corresponding packaged lentiviruses were transduced into primary GBM cells #286 to generate #286-luc. SOX2 and PML were cloned into the BamH I restriction site of the tetracycline-inducible vector pLVX-TetOne-puro (miaolingbio, Wuhan, China), in which SOX2 expression is driven by human EF-1α promoter, while PML expression is Dox -dependent. Lentiviruses were packaged and transduced into U87-luc and #286-luc to express empty vectors (Ctl), SOX2 (-Dox), or SOX2 and PML (+Dox), respectively. U87-luc cells were kindly provided by Prof. Zong-Qiang Cui (Wuhan Institute of Virology).

### Virus and virus infection

HCMV strain Towne (ATCC-VR977) was propagated in HELs as described previously [55]. TB40E was rescued from a bacterial artificial chromosome clone of the HCMV TB40/E strain (a kind gift from Dr. Barbara Adler, Max von Pettenkofer-Institute, Germany) [56], and was propagated for three passages in fibroblasts. The presence of coding sequences of pentamer (gH/gL/UL128-130-131A) were confirmed for the produced virions. In the virus entry assay, cells were infected (MOI = 5) at 4°C to allow HCMV to attach. The unattached virus was removed by washing after 1h incubation, and the culture was moved to 37°C incubator to initiate virus entry. After wash again and trypsinization cells were collected 1h later for IB analysis of cell-associated pp65. For quantitation of viral genome copy number, genomic DNA was extracted from infected cells followed by qPCR to quantitate viral DNAs using HCMV UL83 primers as described [57]. Quantitative reverse transcriptase PCR (qRT-PCR) for examination of HCMV mRNA level were performed as described [58]. Xenograft tumor tissues were homogenized in ice-cold DMEM utilizing a Polytron homogenizer. Viral titers were determined in monolayer HFFs by plaque forming assay.

### Neurosphere formation

Neurosphere formation was assessed with an *in vitro* clonogenic assay [59]. Plasmid pHAGE-SOX2, pHAGE-PML, pHAGE-SOX2-T2A-PML, or pHAGE (Ctl) was transfected into 293T cells along with helper plasmids pSPAX and pMD2G to produce the corresponding lentiviruses. These lentiviruses (expressing GFP as the selection marker) were harvested at 72hpt, titrated in 293T cells by quantifying GFP-positive cells, and stored as aliquots at -80°C for further application. Primary GSCs were transduced with the corresponding lentiviruses at an MOI of 1. Cultures with >90% GFP-positive cells at 5 dpt were harvested, and the expression of the transgene(s) was determined by IB. GSCs transduced with lentiviruses of Ctl, SOX2, and both SOX2 and PML (SOX2/PML) were treated with accutase to single cells and reseeded in 48-well plates (5×10^4^ cells/well). Cells were infected with HCMV Towne or TB40E at an MOI of 5 and treated with ganciclovir (GCV) (150 μM) or untreated (DMSO) for 48 hours. After 3–6 days of incubation, cell cultures were imaged and counted.

### RNA quantification

Total RNA was extracted with Trizol reagent following the manufacturer’s protocols (Invitrogen). Specific mRNAs were quantified by one-step real-time RT-PCR using the QuantiFast SYBR Green RT-PCR kit (Qiagen, Hilden, Germany). The data were normalized to GAPDH expression for each sample. The 2^−ΔΔCt^ method was used to calculate relative expression changes. The primer sequences for quantitative RT-PCR are listed in S8 Table.

### Western blotting

Cells were harvested and lysed with lysis buffer containing protease inhibitor cocktail (Roche), as recommended by the manufacturer. Xenograft tumor tissues of #286-luc and U87-luc from PBS-perfused mice brains were homogenized with lysis buffer. Protein concentrations were determined by Bradford assay (Bio-Rad, WA, USA) and boiled in 5×loading buffer. Cell lysates with equal amounts of total protein were separated by SDS-PAGE, electro-transferred to polyvinylidene difluoride membranes (Millipore), and blocked with 5% nonfat milk solution for 1 h, followed by blotting with primary antibodies. The proteins were visualized using suitable HRP-conjugated secondary antibodies (Jackson Immuno Research, PA, USA) and Super Signal-Femto chemiluminescent substrate (Pierce, TX, USA), and the blots were visualized using Molecular Imager Gel Doc XR System (Bio-Rad). Antibodies used in the study are listed in S6 Table.

### Immunofluorescence analysis (IFA)

Synchronized glioma cells were reseeded onto coverslips and infected with HCMV after attachment. Coverslips were fixed with 4% paraformaldehyde and target proteins were detected by incubation with the primary antibodies and appropriate secondary antibodies (S6 Table) as described previously [60]. Nuclei were counterstained with DAPI (Sigma). For mouse brain tissues, PBS-perfused brains were fixed in 4% paraformaldehyde, and coronal sections were prepared for IFA (thickness: 40 μm). Images were obtained using the Fusion software on a high-speed confocal scanning microscope (Dragonfly 200, ANDOR, Belfast, UK).

### RNA-Seq

Each sample from 3 duplicates of SOX2-overexpressed U251 and Ctl cells was subjected to RNA-Seq. The quality of the raw paired-end reads from RNA-seq were evaluated with FASTQC (www.bioinformatics.babraham.ac.uk/projects/fastqc/). Low-quality reads and contamination were filtered with Trimmomatic [61]. The cleaned reads were aligned to the human genome (Sscrofa10.2) using Burrows-Wheeler Aligner (BWA) [62] and TopHat [63] with default parameters, respectively. Unmapped reads and non-uniquely mapped reads (mapping quality<30) were removed, while PCR duplicate reads were removed using SAMtools [64]. For comprehensively analysis, deepTools2 [65] was used to count the coverage through whole genome with bin size of 1 Mb. The samples were normalized by reads per kilobase per million mapped reads. The normalization of counts and detection of differentially expressed genes (DEGs) were performed by DESeq2 (the absolute value of fold change ≥ 1.5 and P<0.05) [66]. GO analysis was performed using DAVID (42). KEGG pathway enrichment analyses were performed using KOBAS [67]. The top enrichment pathways were visualized using a bar graph showing the -Log10 of FDR corrected p-value. Heatmaps were created using TBtools [68].

### Luciferase reporter assay

To construct the luciferase gene expression vectors, the phosphoglycerate kinase (PGK) promoter of the pmirGLO plasmid was replaced with the *PML* or *Sp100* promoter region, generating pmirGLO-*PML pro-* and pmirGLO-*Sp100 pro*-Luc, respectively. 293T cells in 12-well plates (2.5 × 10^5^ cells/well) were transfected with pmirGLO-*PML pro* or pmirGLO-*Sp100 pro* together with SOX2 or empty control plasmid. pmirGLO-*PML pro-* or pmirGLO-*Sp100 pro*-Luc was transfected in SOX2-OE or Ctl cells of U251 and U87. Luciferase activities were measured 24 h later with Dual-Glo Luciferase Assay System (Promega, WI, USA). Data were normalized by calculating the ratio between firefly luciferase activity and Renilla luciferase activity.

### Animal studies and bioluminescence imaging

Six-week-old BALB/c nu/nu female mice were used and randomized into twelve groups. U87-luc-Ctl, U87-luc-SOX2/PML, #286-luc-Ctl, and #286-luc-SOX2/PML (3 × 10^5^) cells were mock- or HCMV-infected at an MOI of 5. Three hours post infection, cells were digested following a standard operating procedure. Briefly, the cells were washed once with PBS, and exposed to cell digestion solution Accutase (Millipore, SCR005; for #286-luc-Ctl and #286-luc-SOX2/PML) or trypsin (for U87-luc-Ctl and U87-luc-SOX2/PML). When the cells rounded up, the cell digestion solution was replaced with complete cell culture medium. Cells were collected, centrifugated, suspended, and washed twice with PBS. Then the collected cells were suspended in 3μl of PBS and stereotaxically injected into the right striatum of the mice (anterior-posterior (AP) = +0.74 mm, medial-lateral (ML) = +1.68 mm and dorsal-ventral (DV) = -3.5 mm) using a stereotaxic system. Three days before the injection, animals had received drinking water containing doxycycline (Dox, 2 mg/ml enriched with 5% sucrose), whereas control mice had received only sucrose-enriched water. The Dox-containing water was changed every 3 days, and the treatment lasted for one month. GCV were prepared for animal treatment according to the manufacturer’s instructions. GCV was administered once daily (50 mg/kg dose) by intraperitoneal injection. Treatment was begun on day 0 after mice were implanted and continued until day 14 post-implantation. To monitor tumor growth, mice were i.p. injected with 150 mg/kg D-luciferin (APExBIO, TX, USA) and imaged using the IVIS system (Xenogen, CA, USA) 10 min later. The bioluminescence images were analyzed using the Living Image software package version 4.7.3 (Caliper Life Sciences). All animals were monitored closely for signs of neurologic disorders and body weight loss throughout the experiment. Animals were sacrificed when they lost 20% of their body weight or had difficulty feeding, grooming, or ambulating.

### Statistical analysis

Comparisons of the means from different groups were analyzed by two-way ANOVA with the Tukey post-hoc multiple comparisons. Patient survival was analyzed with Cox regression and Kaplan–Meier curves. Two groups of patients were defined according to protein levels and compared by the log-rank test. Differences in HCMV IE1 and SOX2 expression based on IHC among different groups were analyzed by one-way ANOVA. Correlation between SOX2 and PML or SOX2 and Sp100 expression in glioma samples was analyzed by Crosstabs. Statistical analyses were conducted using Prism Graphpad (version 8.43). Differences were considered statistically significant when p≤0.05.

## Supporting information

S1 Fig HCMV IE1 expression correlates with SOX2 level in glioma cells. (**A**) Glioma cells of U251, U87, LN229, T98G, A172 and CCF were mock- (M) or HCMV Towne strain-infected (V) at an MOI of 5. (**A**) SOX2 and HCMV IE1 expression in glioma cell lines. Quantification of SOX2-positive and IE1-positive cells is shown in (**B**) and (**C**), respectively. Data are from three independent experiments and represent as means ±SEM.
(TIF)

S2 Fig SOX2-OE promotes HCMV gene transcription. SOX2 overexpressing (SOX2-OE) and control (Ctl) cells were generated from U251, U87, LN229, T98G, A172 and CCF cells by lentivirus transduction. The cells were infected with HCMV Towne strain at an MOI of 5 and collected at the indicated times for viral gene transcription quantification. mRNA levels of HCMV genes (*UL123*, *UL44* and *UL99*) in SOX2-OE and Ctl cells were determined by RT-qPCR. Data are from three independent experiments and represent as means ±SEM.
(TIF)

S3 Fig SOX2-KO decreases HCMV gene transcription. SOX2 knockout (SOX2 -KO) and negative control (NC) cells were generated from U251, LN229, and CCF cells by lentivirus transduction. The cells were infected with HCMV Towne strain at an MOI of 5 and collected at the indicated times for viral gene transcription quantification. mRNA levels of HCMV genes (*UL123*, *UL44* and *UL99*) in SOX2-KO and NC cells were determined by RT-qPCR. Data are from three independent experiments and represent as means ±SEM.
(TIF)

S4 Fig SOX2 KO inhibits HCMV gene expression in glioma cells. By lentivirus transduction, SOX2 knockout (KO) and negative control (NC) cells were made from U251, LN229 and CCF cell lines and two primary GSCs, #286 and #352. The cells were infected with the HCMV Towne strain at an MOI of 5 and collected at the indicated times for IB analysis. SOX2 protein and HCMV proteins of IE1/2, UL44, and pp28 in SOX2-KO and NC cells are shown. Data are from three independent experiments. GAPDH served as an internal control for protein quantification normalization. The numbers below the blot indicate relative levels of the indicated proteins to those in NC cells at 24 hpi. The GSC-KO cells were not subjected to single-cell clone selection and thus represented a heterogeneous cell population.
(TIF)

S5 Fig Effect of SOX2 on HCMV entry, replication and virion release. SOX2 overexpressing (SOX2-OE) and control (Ctl) cells based on glioma cell lines U251 and U87, and primary GSCs #286 and #352 were infected with HCMV Towne strain at an MOI of 5. Cells were harvested at the indicated times for analyses of entry, viral genome replication, and infectious virus titers. (**A**) Viral entry determined by input pp65 level. Cells were harvested at 1 hpi and analyzed by IB to detect pp65. (**B**) Viral replication. The cells were plated onto coverslips and the expression of SOX2 and UL44 was examined by IFA. UL44 positive cells were quantified. (**C**) Viral genome level. Plasmids pcDNA3.0-UL83 and pcDNA3.0-GAPDH were used to generate standard curves as previously described [57]. HCMV genome copy numbers were determined by qRT-PCR and standardized to cellular DNA (GAPDH) copy number to produce viral genome copies/cell. (**D**) Viral titer. Infectious viruses in the culture supernatants at the indicated times were determined by plaque forming assay. Data are from three independent experiments and represent as means ±SEM (One-way ANOVA; *, p < 0.05; **, p < 0.01).
(TIF)

S6 Fig Effects of SOX2 on the HCMV major immediate early promoter (MIEP) activities. To construct the luciferase gene expression vectors, the phosphoglycerate kinase (PGK) promoter of the pmirGLO plasmid was replaced with the MIEP enhancer and promoter sequence form the plasmid of pHAGE, generating pmirGLO-MIEP pro-luc. 293T cells in 12-well plates (2.5 × 10^5^ cells/well) were transfected with pmirGLO-MIEP pro-luc together with SOX2 expressing or empty control plasmid. pmirGLO-MIEP pro-luc was transfected in SOX2-OE or Ctl cells of U251 and U87. Luciferase activities were measured 24 h later with Dual-Glo Luciferase Assay System (Promega, WI, USA). Data were normalized by calculating the ratio between firefly luciferase activity and Renilla luciferase activity. Data are from three independent experiments and represent as means ±SEM.
(TIF)

S7 Fig PML inhibits HCMV gene transcription. PML knockout (PML-KO), PML overexpressing (PML-OE), and their controls (Ctl and NC, respectively) cells were infected with HCMV Towne strain at an MOI of 5 and collected at the indicated times for viral gene transcription quantification. mRNA levels of HCMV genes (*UL123*, *UL44*, and *UL99*) in PML-KO and NC (**A**), as well as PML-OE and Ctl (**B**) cells, were determined by RT-qPCR. Data are from three independent experiments and represent as means ±SEM.
(TIF)

S8 Fig Sp100 inhibits HCMV gene transcription. Sp100 knockout (Sp100-KO), Sp100 overexpressing (Sp100-OE), and their controls (Ctl and NC, respectively) cells were infected with HCMV Towne strain at an MOI of 5 and collected at the indicated times for viral gene transcription quantification. mRNA levels of HCMV genes (*UL123*, *UL44*, and *UL99*) in Sp100-KO and NC (**A**), as well as Sp100-OE and Ctl (**B**) cells, were determined by RT-qPCR. Data are from three independent experiments and represent as means ±SEM.
(TIF)

S9 Fig SOX2 promotes HCMV infection *in vivo*. Protein levels of SOX2, PML, and HCMV IE1/2 in the tumors tissue of xenograft mice bearing mock- (M) or HCMV Towne strain-infected (V) cells at week 2 post implantation. Representative images of (**A**) #286-cell and (**B**) U87-cell bearing mice are shown. GAPDH served as a loading control.
(TIF)

S10 Fig SOX2 accelerates tumor growth in U87-luc bearing mice. Bioluminescence images (**A**) and quantification (**B**) of luciferase-expressing tumors in nude mice (n = 7 for each group) bearing mock- (M) or HCMV Towne strain -infected (V) U87-luc-Ctl, U87-luc-SOX2/PML (-Dox) and U87-luc-SOX2/PML (+Dox) cells. (**C**) Four weeks after tumor cell implantation, coronal sections of U87-luc tumor-bearing mouse brains treated in different groups were stained with H&E. Representative images of each group are presented. Representative images and quantification of immunostaining for Ki-67 (green) (**D**) and PDGFD (green) (**E**) in the tumors. DAPI-stained nuclei are shown in blue. Data were collected from 5 images/mice and n = 3 mice/group. Data in (**B**, **D** and **E**) are means ±SEM (Two-way ANOVA along with the Tukey post-hoc multiple comparisons, the p-values and statistical parameters are provided in Tables H-J in S1 Table).
(TIF)

S11 Fig Quantitative analysis of angiogenesis and vessel morphology. Representative images and quantification of CD31 immunostaining (green) in the tumors of #286-luc (**A**) and U87-luc (**B**) xenografts. Data were collected from 5 images/mice and n = 3 mice/group. Data are means ±SEM (Two-way ANOVA along with the Tukey post-hoc multiple comparisons, the p-values and statistical parameters are provided in Tables K and L in S1 Table).
(TIF)

S12 Fig Effects of SOX2 overexpression and HCMV TB40E on neurosphere formation of GSCs. Patient-derived GSCs (#286 and #352) were transduced with SOX2 expressing (SOX2) or control lentivirus (Ctl). The transduced GSCs cultured in 48-well plates (2 x 10^5^/well) were mock- (M) or TB40E-infected (TB40E) at an MOI of 5. Cells in a few wells were treated with 150 μM ganciclovir (GCV) for 48 hours. Representative images of neurospheres formed by GSC #286 (**A**) and GSC #352 (**B**) are shown. Quantification of neurospheres (number/field and size) and total cells at 144 hpi is shown in (**C-E**) for GSC #286 and (**F-H**) for GSC #352. For each condition, three wells (five random fields/well) were employed for neurosphere quantification, and cell counting was performed after digestion of neurospheres into a single cell for each well. Data are from three independent experiments and presented as means ±SEM. Two-way ANOVA analyses along with the Tukey post-hoc multiple comparisons were performed to evaluate the statistically significant differences between groups (**C-H**), and the p-values and statistical parameters are provided in Tables M-R in S1 Table. (**I**) The mock- or TB40E-infected neurospheres of GSC #286-Ctl and -SOX2 OE at 144 hpi were fixed and stained with DAPI. Representative images are shown.
(TIF)

S13 Fig SOX2 enhances the oncomodulatory activities of HCMV TB40E in a patient-derived xenograft GBM model. Patient-derived #286-luc-Ctl (Ctl) and #286-SOX2 cells were mock- (M) or TB40E-infected (TB40E) for three hours and then implanted into the brains of nude mice using a stereotaxic system. The tumor growth was monitored at week 2 and week 4 (**A**) and quantified by signal intensity (**B**). (**C**) Brain tissues were stained with H&E at week 4 post implantation. (**D**) Survival curves of #286-luc tumor-bearing mice of different groups. N = 5 mice/group. The log-rank test was used to compare animal survival. Representative images and quantification of Ki-67 (**E**) and PDGFD (**F**) immunostaining (green) in the tumors at week 4 are shown. Data were collected from 5 images/mice and n = 3 mice/group. Data in (**B**, **E** and **F**) represent as means ±SEM (Two-way ANOVA along with the Tukey post-hoc multiple comparisons, the p-values and statistical parameters are provided Tables S-U in S1 Table).
(TIF)

S14 Fig HCMV IE1 and pp65 expression in tumor tissues from two GBM cases (#318 and #337). IE1 and pp65 expression in tumor tissues of GBM cases #318 and #337 were tested by IHC. Representative images are shown.
(TIF)

S15 Fig Expression of PML-NB components and HCMV IE1/2 in glioma cells. The cells were mock- (M), HCMV Towne strain- or TB40E-infected (V) at an MOI of 1 (for HFFs infection) or 5 (for glioma cell infection) and collected at the indicated times for IB analysis of IE1/2, PML, Sp100, Daxx, and SOX2. HFFs (**A**), primary glioblastoma cells #286, and #352 (**B**) were infected with TB40E. U251 and U87 (**C**) were infected with the Towne strain. GAPDH served as a loading control. The numbers below the blots indicate relative levels of the indicated proteins to those in mock-infected cells at 24 hpi.
(TIF)

S1 Table Two-way ANOVA analysis used in this study. (XLSX)

S2 Table Categorical Variable Codings^a^ used in Kaplan-Meier curves shown in Fig 7K. (DOCX)

S3 Table Variables used in Cox regression analysis to consider association between patient prognosis and IE1/SOX2 levels. (DOCX)

S4 Table Assigned coefficients of variables in Cox Regression analysis in Fig 7K. (DOCX)

S5 Table Characteristics of patients involved in this study. (DOCX)

S6 Table Antibodies used in this study. (DOCX)

S7 Table SgRNA sequences used in this study. (DOCX)

S8 Table qPCR Primers used in this study. (DOCX)
