# Supplementary figures and images for: SOX2 downregulation of PML increases HCMV gene expression and growth of glioma cells
Source: PLoS Pathog. 2023 Apr 14;19(4):e1011316. doi: 10.1371/journal.ppat.1011316 (PMC10104302; doi:10.1371/journal.ppat.1011316)

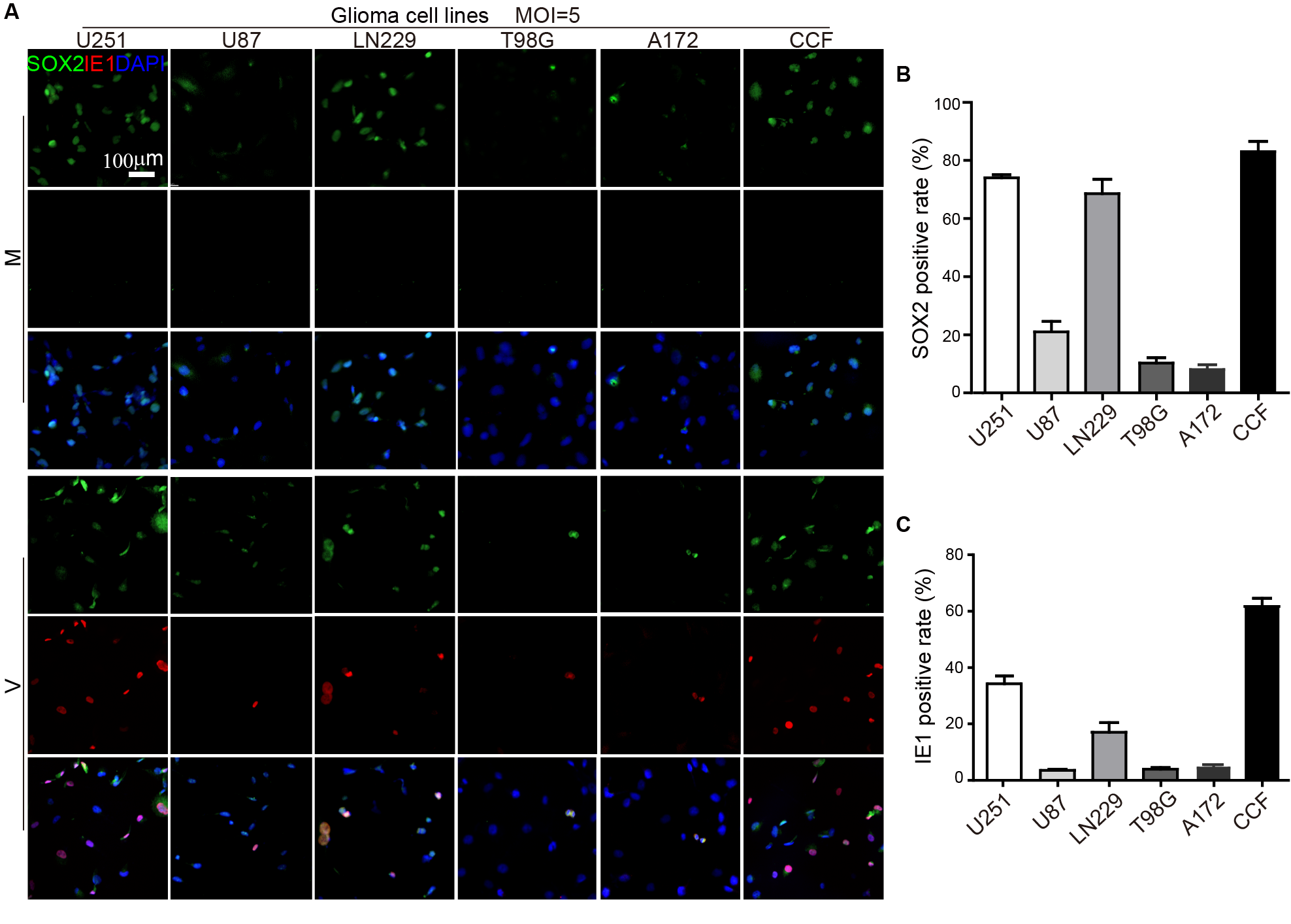

Supplement: S1 Fig — (A) Glioma cells of U251, U87, LN229, T98G, A172 and CCF were mock- (M) or HCMV Towne strain-infected (V) at an MOI of 5. (A) SOX2 and HCMV IE1 expression in glioma cell lines. Quantification of SOX2-positive and IE1-positive cells is shown in (B) and (C), respectively. Data are from three independent experiments and represent as means ±SEM. (TIF) [file ppat.1011316.s001.tif]

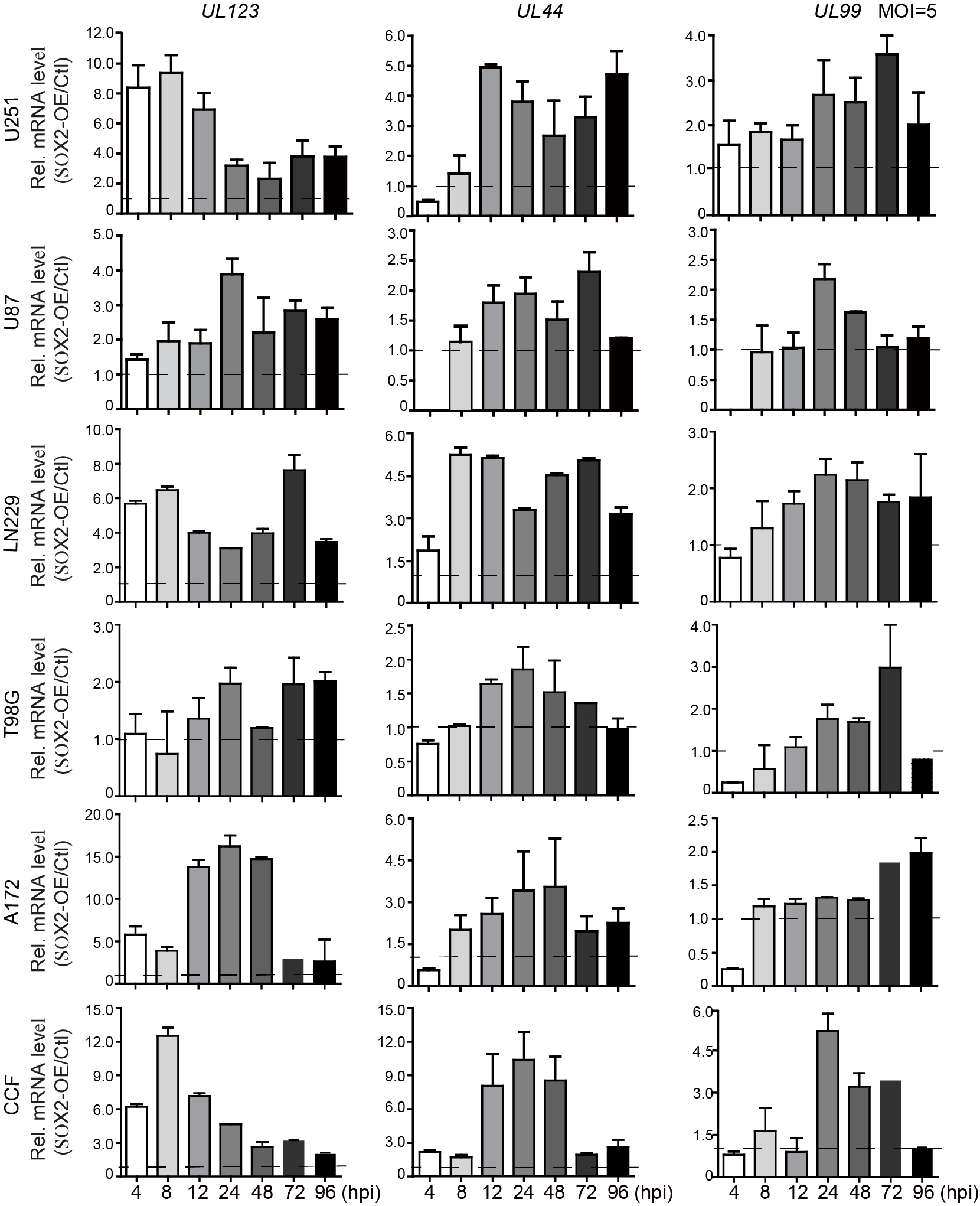

Supplement: S2 Fig — SOX2 overexpressing (SOX2-OE) and control (Ctl) cells were generated from U251, U87, LN229, T98G, A172 and CCF cells by lentivirus transduction. The cells were infected with HCMV Towne strain at an MOI of 5 and collected at the indicated times for viral gene transcription quantification. mRNA levels of HCMV genes (UL123, UL44 and UL99) in SOX2-OE and Ctl cells were determined by RT-qPCR. Data are from three independent experiments and represent as means ±SEM. (TIF) [file ppat.1011316.s002.tif]

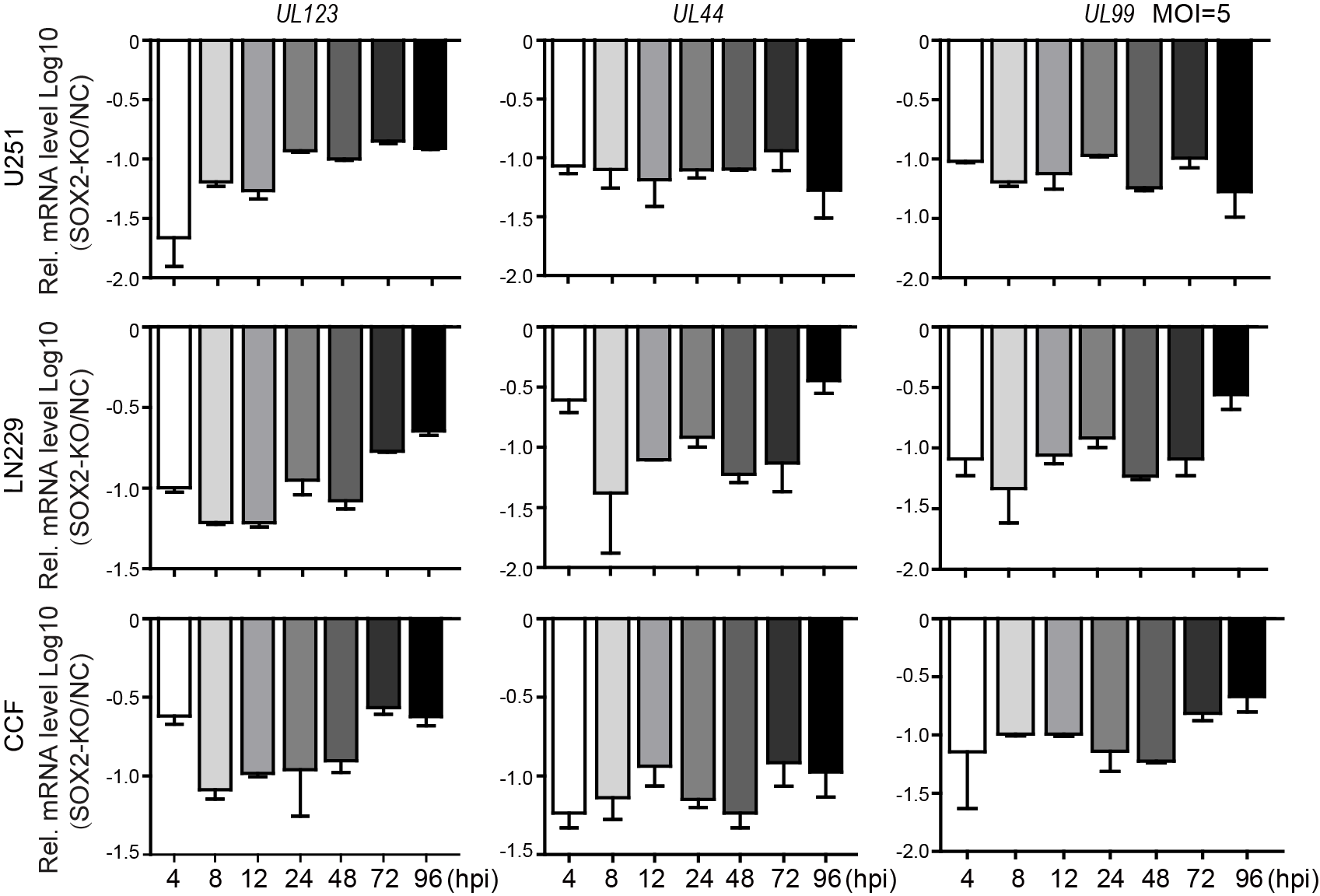

Supplement: S3 Fig — SOX2 knockout (SOX2 -KO) and negative control (NC) cells were generated from U251, LN229, and CCF cells by lentivirus transduction. The cells were infected with HCMV Towne strain at an MOI of 5 and collected at the indicated times for viral gene transcription quantification. mRNA levels of HCMV genes (UL123, UL44 and UL99) in SOX2-KO and NC cells were determined by RT-qPCR. Data are from three independent experiments and represent as means ±SEM. (TIF) [file ppat.1011316.s003.tif]

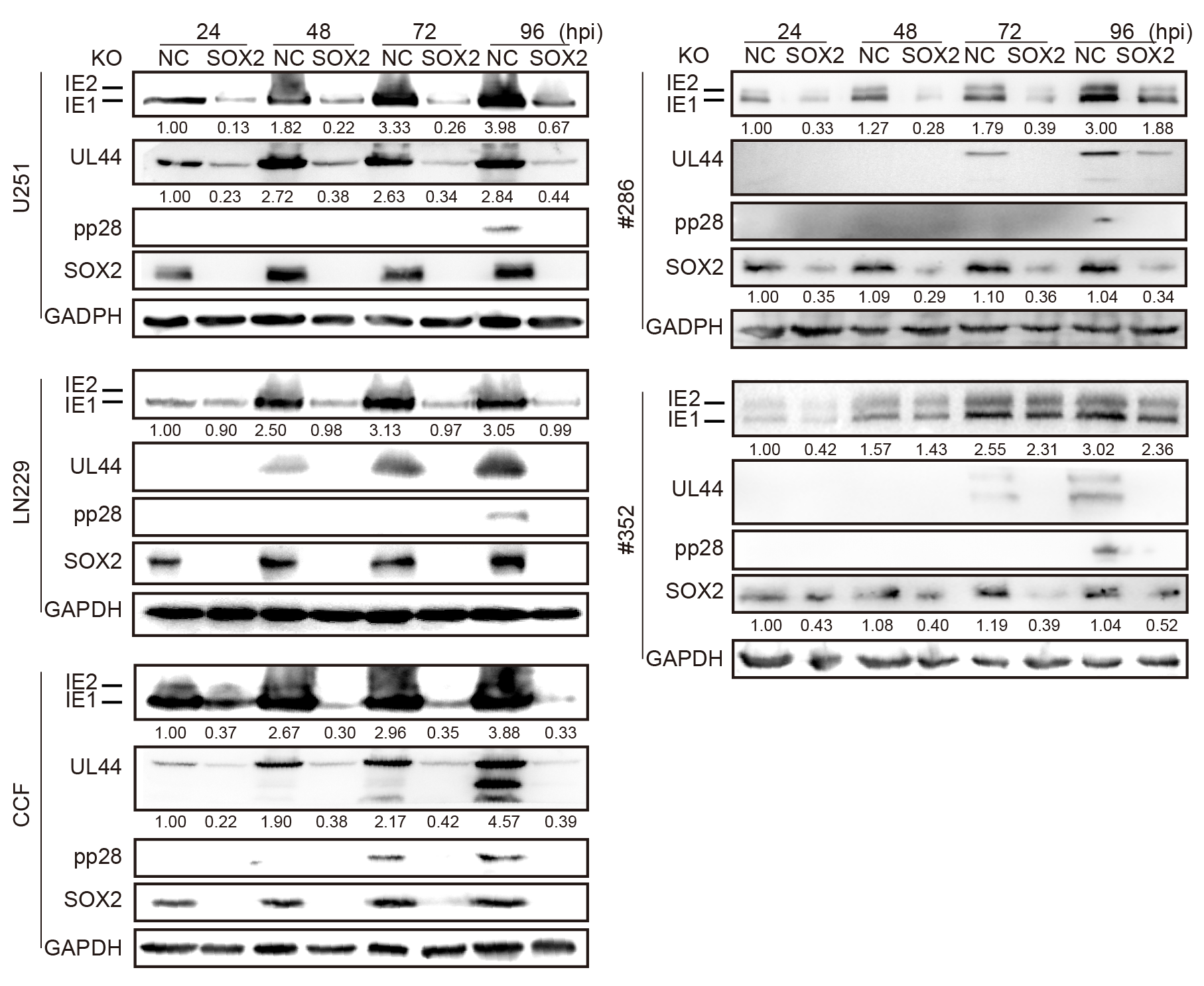

Supplement: S4 Fig — By lentivirus transduction, SOX2 knockout (KO) and negative control (NC) cells were made from U251, LN229 and CCF cell lines and two primary GSCs, #286 and #352. The cells were infected with the HCMV Towne strain at an MOI of 5 and collected at the indicated times for IB analysis. SOX2 protein and HCMV proteins of IE1/2, UL44, and pp28 in SOX2-KO and NC cells are shown. Data are from three independent experiments. GAPDH served as an internal control for protein quantification normalization. The numbers below the blot indicate relative levels of the indicated proteins to those in NC cells at 24 hpi. The GSC-KO cells were not subjected to single-cell clone selection and thus represented a heterogeneous cell population. (TIF) [file ppat.1011316.s004.tif]

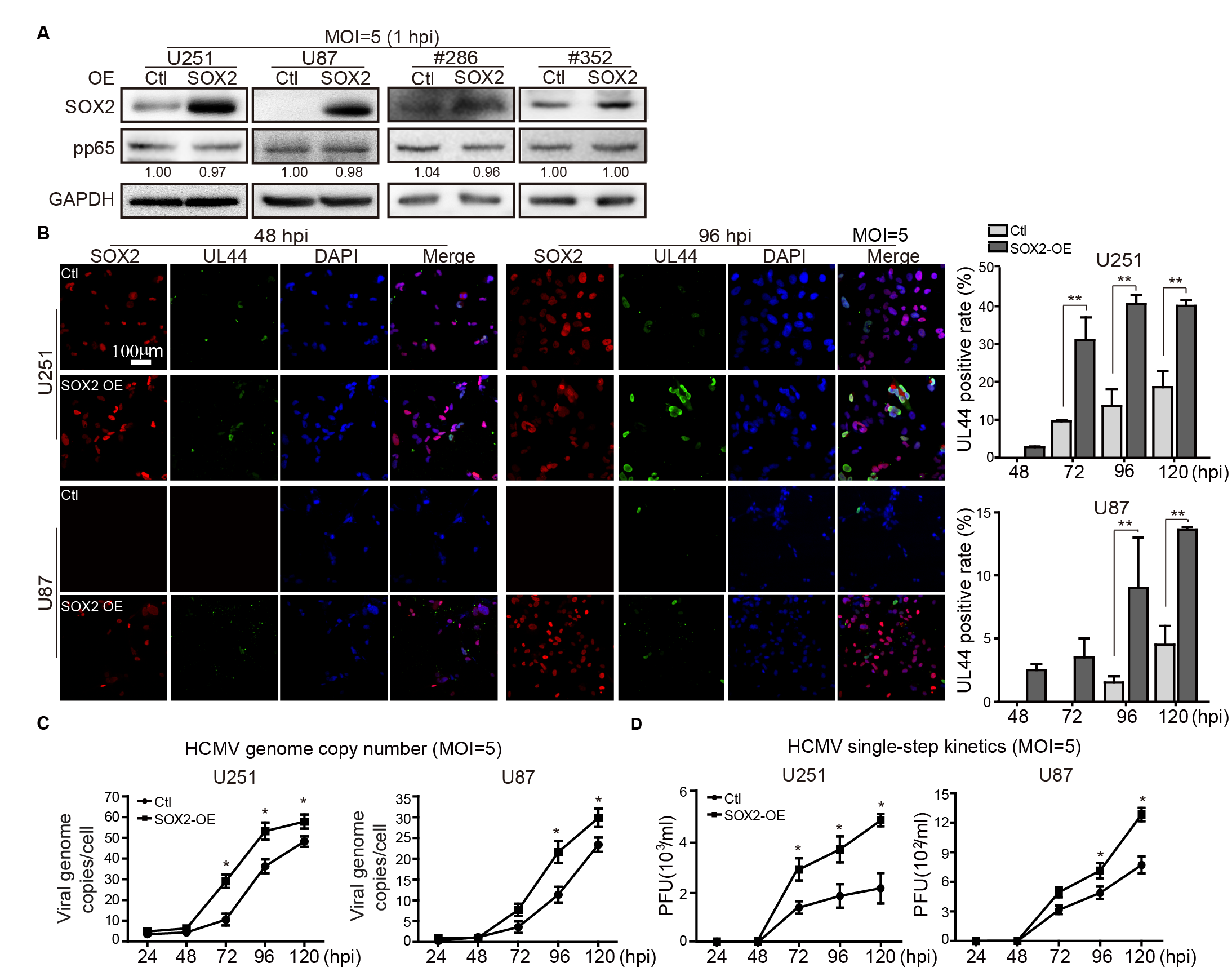

Supplement: S5 Fig — SOX2 overexpressing (SOX2-OE) and control (Ctl) cells based on glioma cell lines U251 and U87, and primary GSCs #286 and #352 were infected with HCMV Towne strain at an MOI of 5. Cells were harvested at the indicated times for analyses of entry, viral genome replication, and infectious virus titers. (A) Viral entry determined by input pp65 level. Cells were harvested at 1 hpi and analyzed by IB to detect pp65. (B) Viral replication. The cells were plated onto coverslips and the expression of SOX2 and UL44 was examined by IFA. UL44 positive cells were quantified. (C) Viral genome level. Plasmids pcDNA3.0-UL83 and pcDNA3.0-GAPDH were used to generate standard curves as previously described [57]. HCMV genome copy numbers were determined by qRT-PCR and standardized to cellular DNA (GAPDH) copy number to produce viral genome copies/cell. (D) Viral titer. Infectious viruses in the culture supernatants at the indicated times were determined by plaque forming assay. Data are from three independent experiments and represent as means ±SEM (One-way ANOVA; *, p < 0.05; **, p < 0.01). (TIF) [file ppat.1011316.s005.tif]

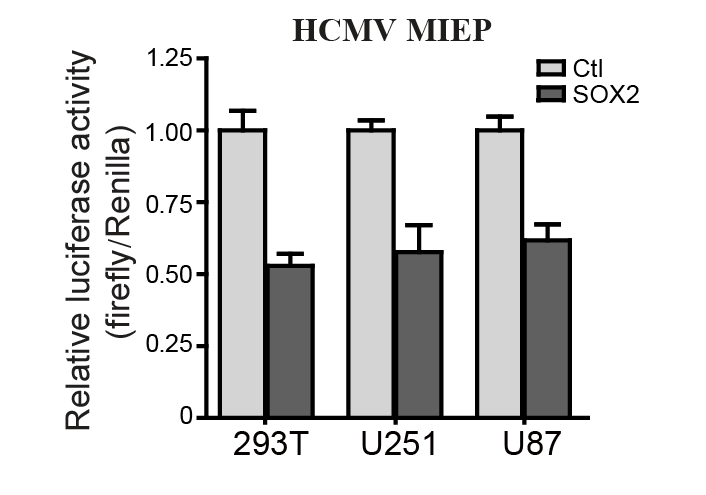

Supplement: S6 Fig — To construct the luciferase gene expression vectors, the phosphoglycerate kinase (PGK) promoter of the pmirGLO plasmid was replaced with the MIEP enhancer and promoter sequence form the plasmid of pHAGE, generating pmirGLO-MIEP pro-luc. 293T cells in 12-well plates (2.5 × 10^5^ cells/well) were transfected with pmirGLO-MIEP pro-luc together with SOX2 expressing or empty control plasmid. pmirGLO-MIEP pro-luc was transfected in SOX2-OE or Ctl cells of U251 and U87. Luciferase activities were measured 24 h later with Dual-Glo Luciferase Assay System (Promega, WI, USA). Data were normalized by calculating the ratio between firefly luciferase activity and Renilla luciferase activity. Data are from three independent experiments and represent as means ±SEM. (TIF) [file ppat.1011316.s006.tif]

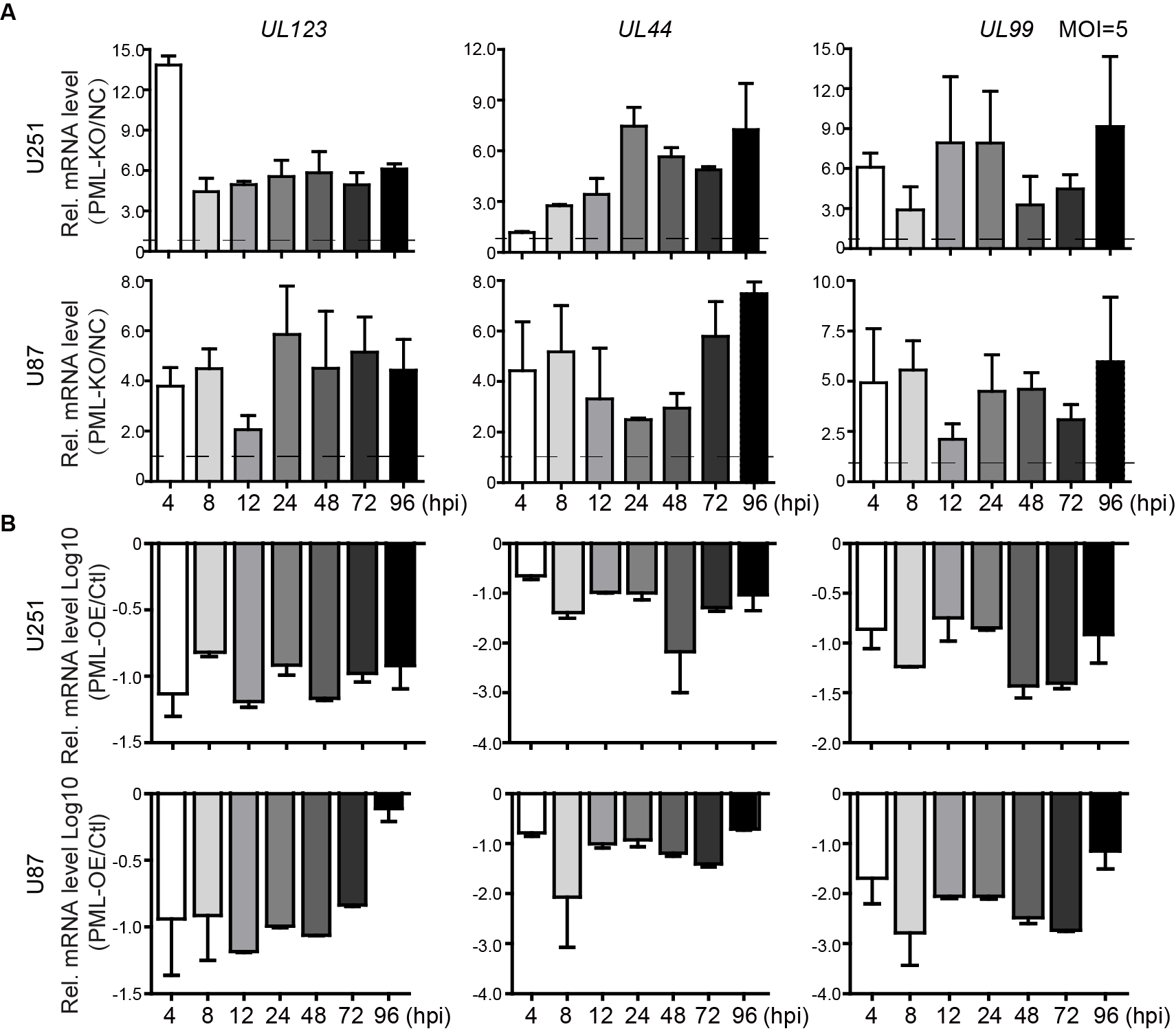

Supplement: S7 Fig — PML knockout (PML-KO), PML overexpressing (PML-OE), and their controls (Ctl and NC, respectively) cells were infected with HCMV Towne strain at an MOI of 5 and collected at the indicated times for viral gene transcription quantification. mRNA levels of HCMV genes (UL123, UL44, and UL99) in PML-KO and NC (A), as well as PML-OE and Ctl (B) cells, were determined by RT-qPCR. Data are from three independent experiments and represent as means ±SEM. (TIF) [file ppat.1011316.s007.tif]

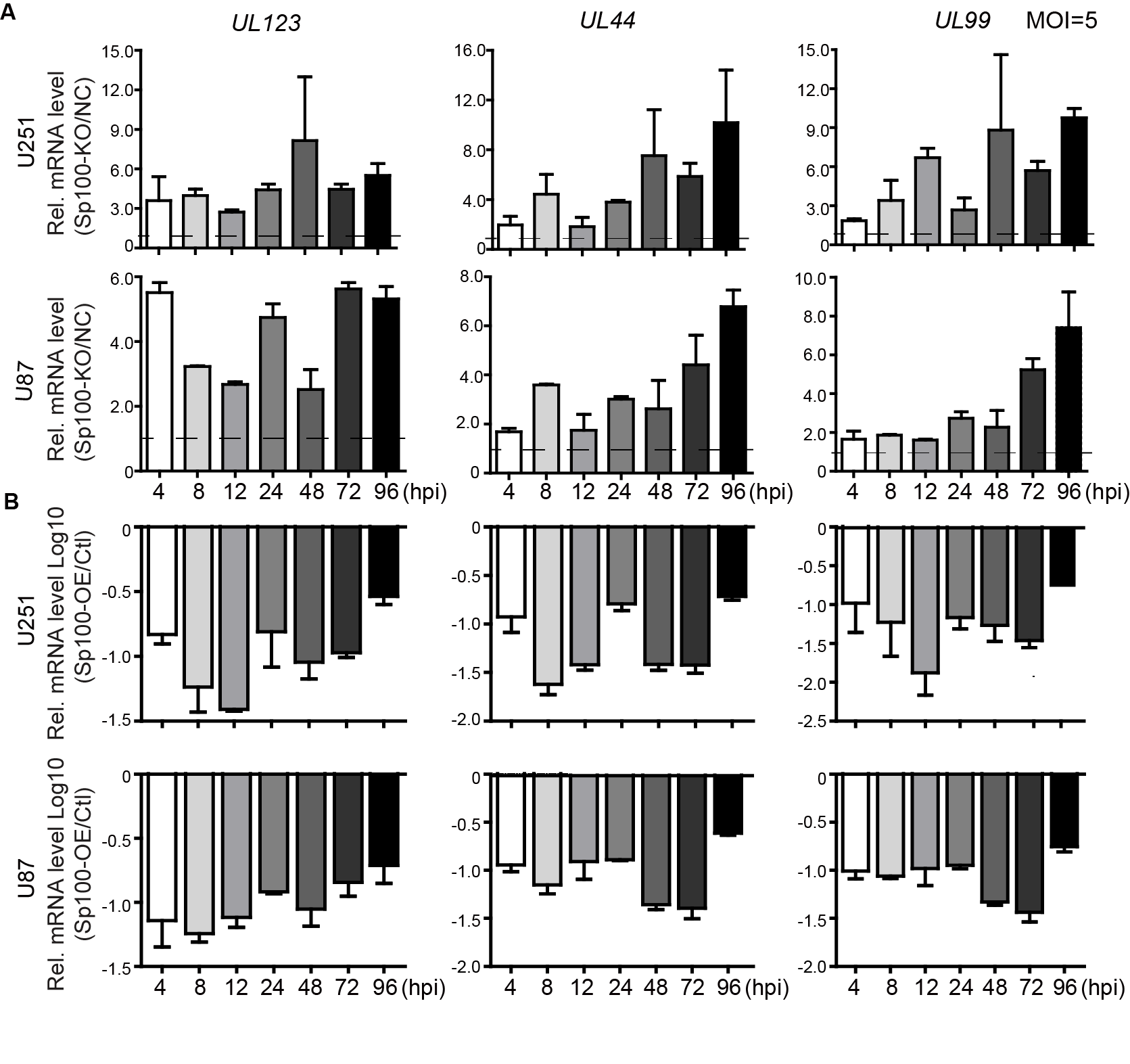

Supplement: S8 Fig — Sp100 knockout (Sp100-KO), Sp100 overexpressing (Sp100-OE), and their controls (Ctl and NC, respectively) cells were infected with HCMV Towne strain at an MOI of 5 and collected at the indicated times for viral gene transcription quantification. mRNA levels of HCMV genes (UL123, UL44, and UL99) in Sp100-KO and NC (A), as well as Sp100-OE and Ctl (B) cells, were determined by RT-qPCR. Data are from three independent experiments and represent as means ±SEM. (TIF) [file ppat.1011316.s008.tif]

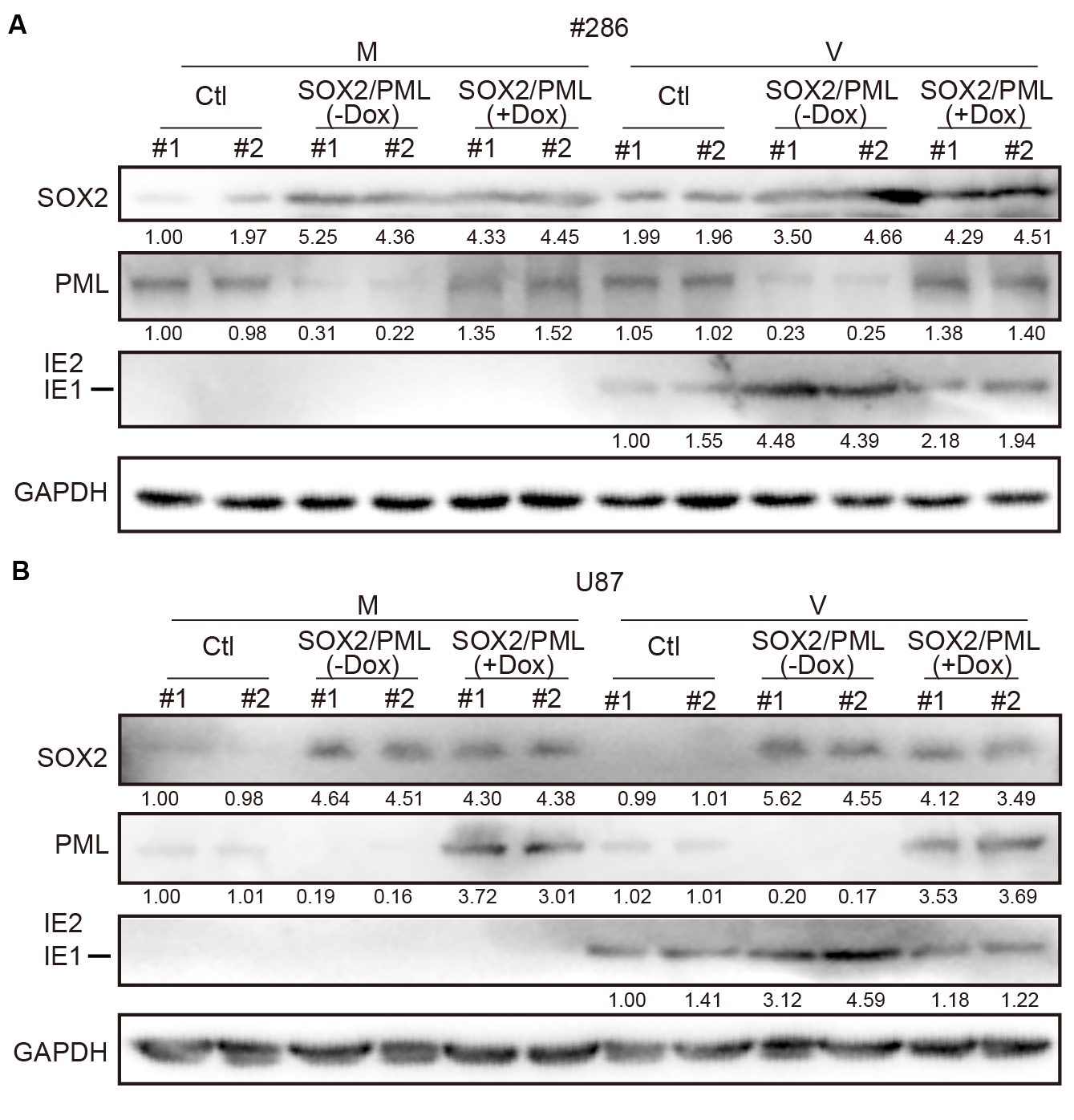

Supplement: S9 Fig — Protein levels of SOX2, PML, and HCMV IE1/2 in the tumors tissue of xenograft mice bearing mock- (M) or HCMV Towne strain-infected (V) cells at week 2 post implantation. Representative images of (A) #286-cell and (B) U87-cell bearing mice are shown. GAPDH served as a loading control. (TIF) [file ppat.1011316.s009.tif]

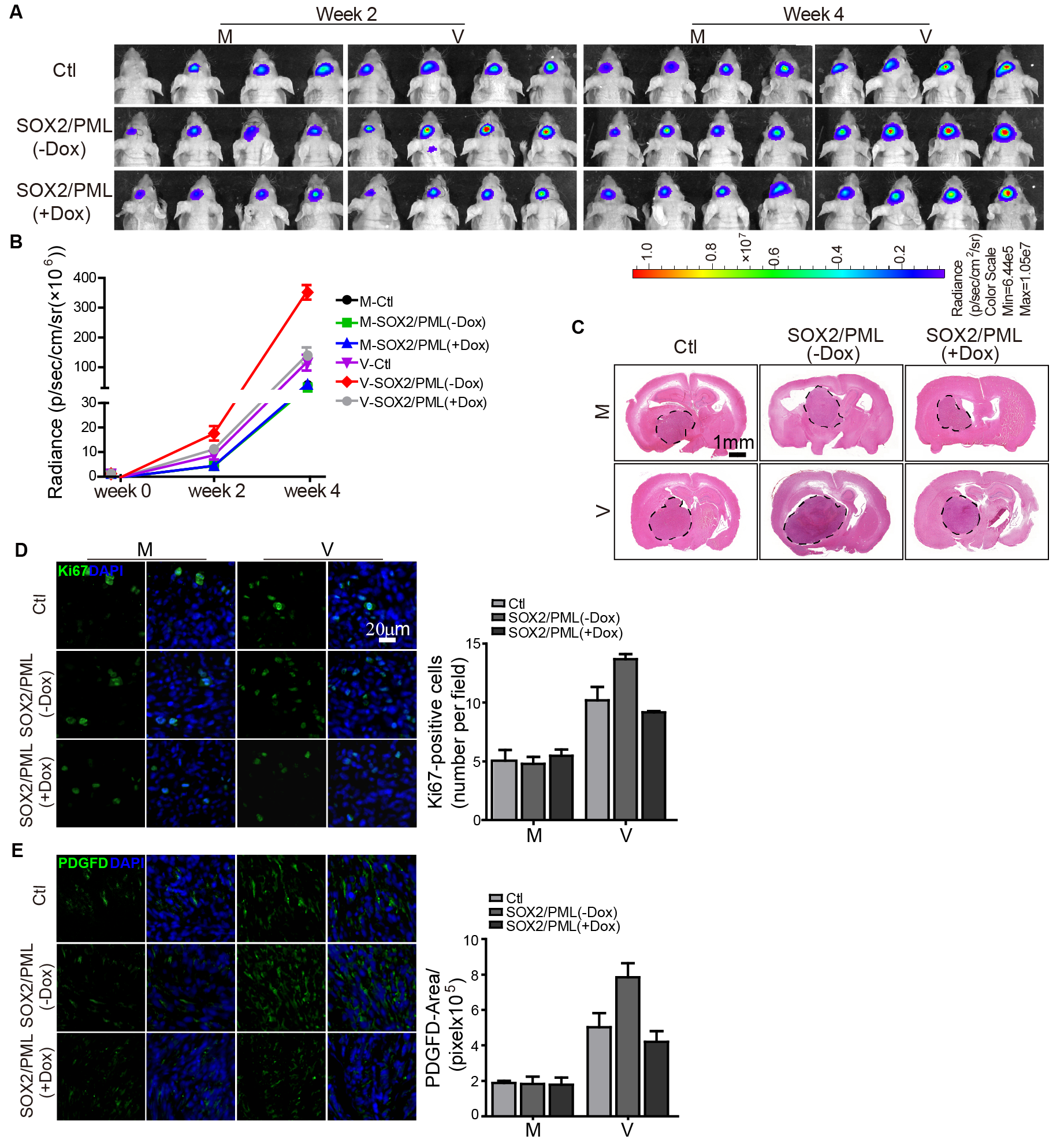

Supplement: S10 Fig — Bioluminescence images (A) and quantification (B) of luciferase-expressing tumors in nude mice (n = 7 for each group) bearing mock- (M) or HCMV Towne strain -infected (V) U87-luc-Ctl, U87-luc-SOX2/PML (-Dox) and U87-luc-SOX2/PML (+Dox) cells. (C) Four weeks after tumor cell implantation, coronal sections of U87-luc tumor-bearing mouse brains treated in different groups were stained with H&E. Representative images of each group are presented. Representative images and quantification of immunostaining for Ki-67 (green) (D) and PDGFD (green) (E) in the tumors. DAPI-stained nuclei are shown in blue. Data were collected from 5 images/mice and n = 3 mice/group. Data in (B, D and E) are means ±SEM (Two-way ANOVA along with the Tukey post-hoc multiple comparisons, the p-values and statistical parameters are provided in Tables H-J in S1 Table). (TIF) [file ppat.1011316.s010.tif]

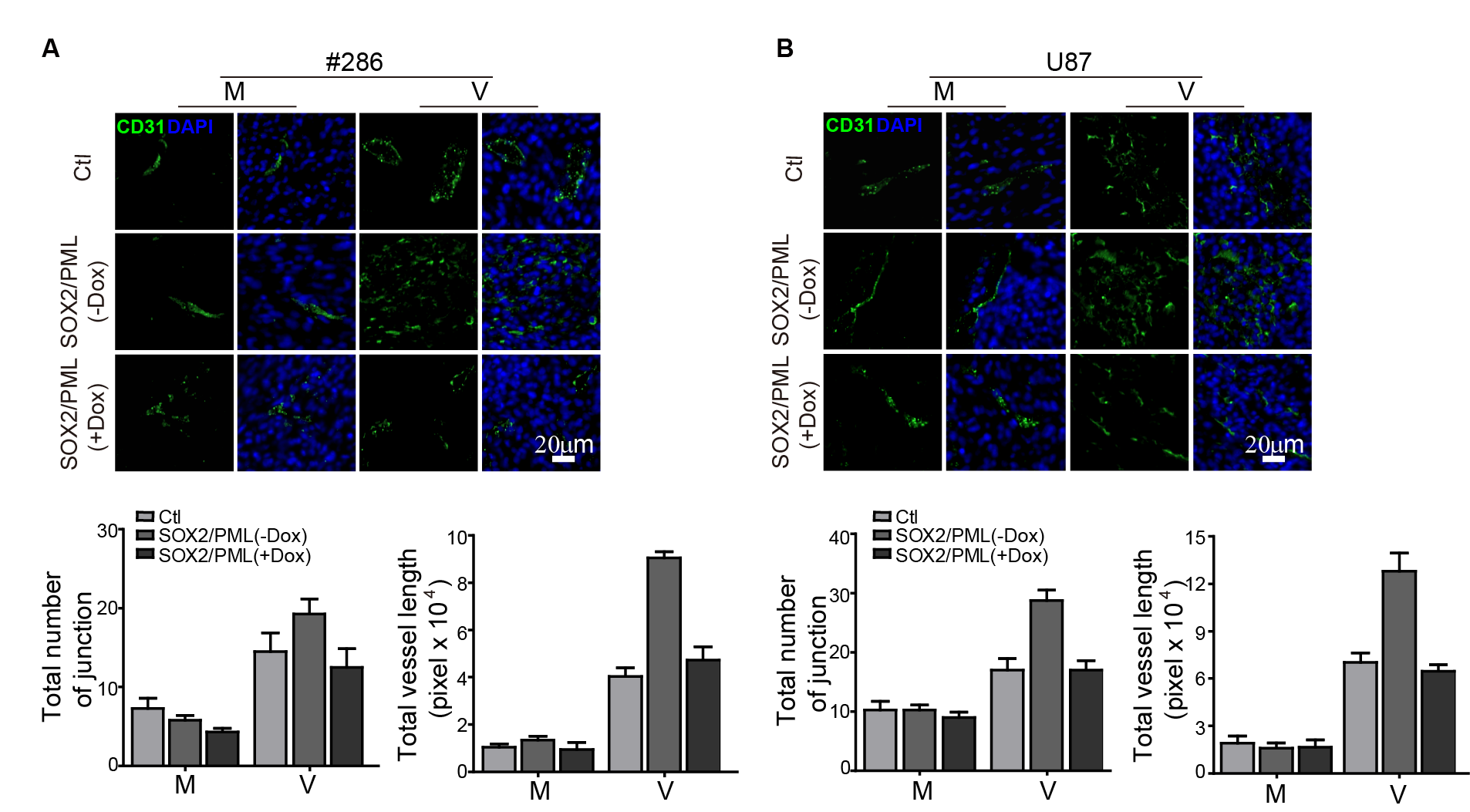

Supplement: S11 Fig — Representative images and quantification of CD31 immunostaining (green) in the tumors of #286-luc (A) and U87-luc (B) xenografts. Data were collected from 5 images/mice and n = 3 mice/group. Data are means ±SEM (Two-way ANOVA along with the Tukey post-hoc multiple comparisons, the p-values and statistical parameters are provided in Tables K and L in S1 Table). (TIF) [file ppat.1011316.s011.tif]

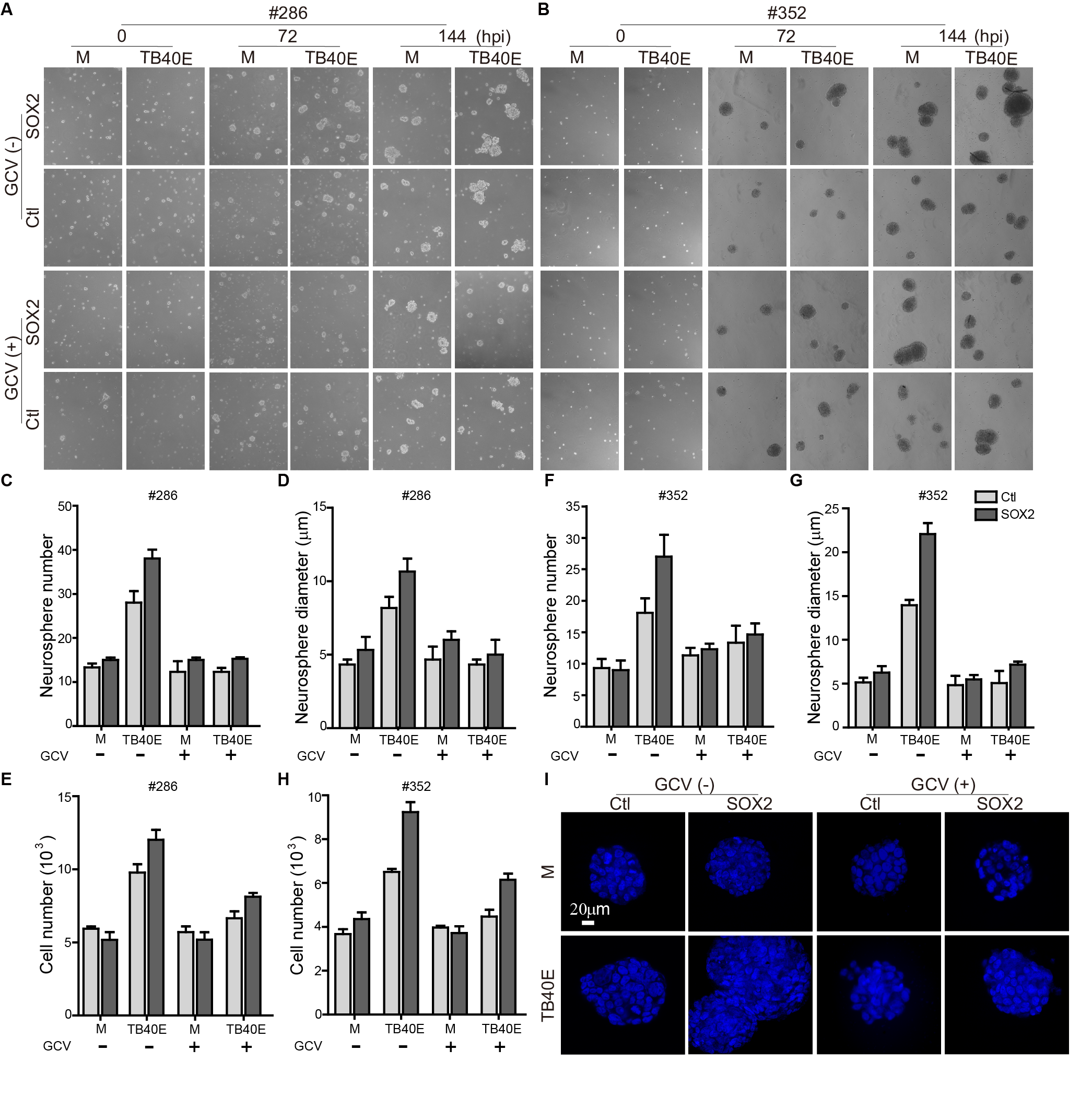

Supplement: S12 Fig — Patient-derived GSCs (#286 and #352) were transduced with SOX2 expressing (SOX2) or control lentivirus (Ctl). The transduced GSCs cultured in 48-well plates (2 x 10^5^/well) were mock- (M) or TB40E-infected (TB40E) at an MOI of 5. Cells in a few wells were treated with 150 μM ganciclovir (GCV) for 48 hours. Representative images of neurospheres formed by GSC #286 (A) and GSC #352 (B) are shown. Quantification of neurospheres (number/field and size) and total cells at 144 hpi is shown in (C-E) for GSC #286 and (F-H) for GSC #352. For each condition, three wells (five random fields/well) were employed for neurosphere quantification, and cell counting was performed after digestion of neurospheres into a single cell for each well. Data are from three independent experiments and presented as means ±SEM. Two-way ANOVA analyses along with the Tukey post-hoc multiple comparisons were performed to evaluate the statistically significant differences between groups (C-H), and the p-values and statistical parameters are provided in Tables M-R in S1 Table. (I) The mock- or TB40E-infected neurospheres of GSC #286-Ctl and -SOX2 OE at 144 hpi were fixed and stained with DAPI. Representative images are shown. (TIF) [file ppat.1011316.s012.tif]

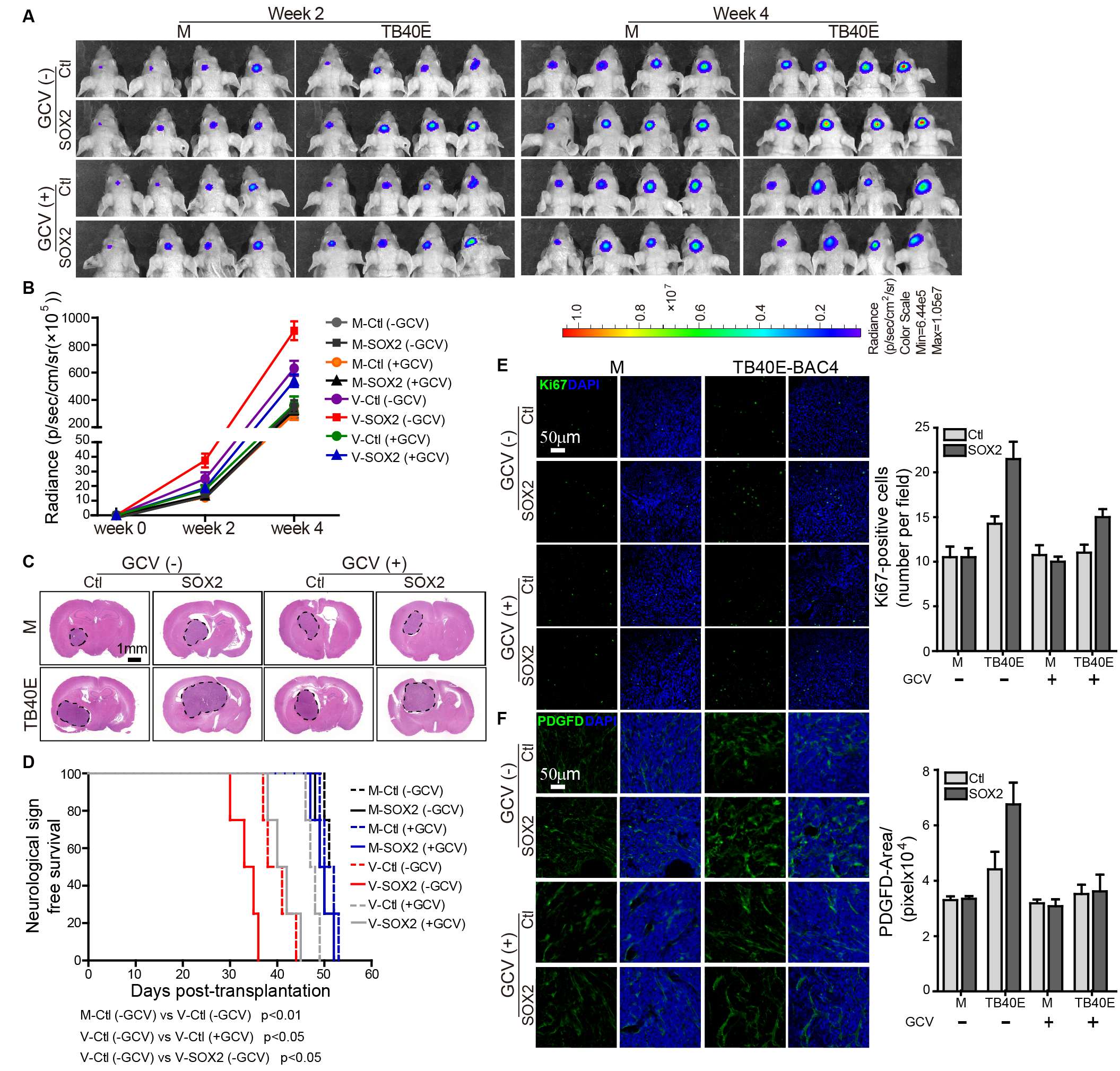

Supplement: S13 Fig — Patient-derived #286-luc-Ctl (Ctl) and #286-SOX2 cells were mock- (M) or TB40E-infected (TB40E) for three hours and then implanted into the brains of nude mice using a stereotaxic system. The tumor growth was monitored at week 2 and week 4 (A) and quantified by signal intensity (B). (C) Brain tissues were stained with H&E at week 4 post implantation. (D) Survival curves of #286-luc tumor-bearing mice of different groups. N = 5 mice/group. The log-rank test was used to compare animal survival. Representative images and quantification of Ki-67 (E) and PDGFD (F) immunostaining (green) in the tumors at week 4 are shown. Data were collected from 5 images/mice and n = 3 mice/group. Data in (B, E and F) represent as means ±SEM (Two-way ANOVA along with the Tukey post-hoc multiple comparisons, the p-values and statistical parameters are provided Tables S-U in S1 Table). (TIF) [file ppat.1011316.s013.tif]

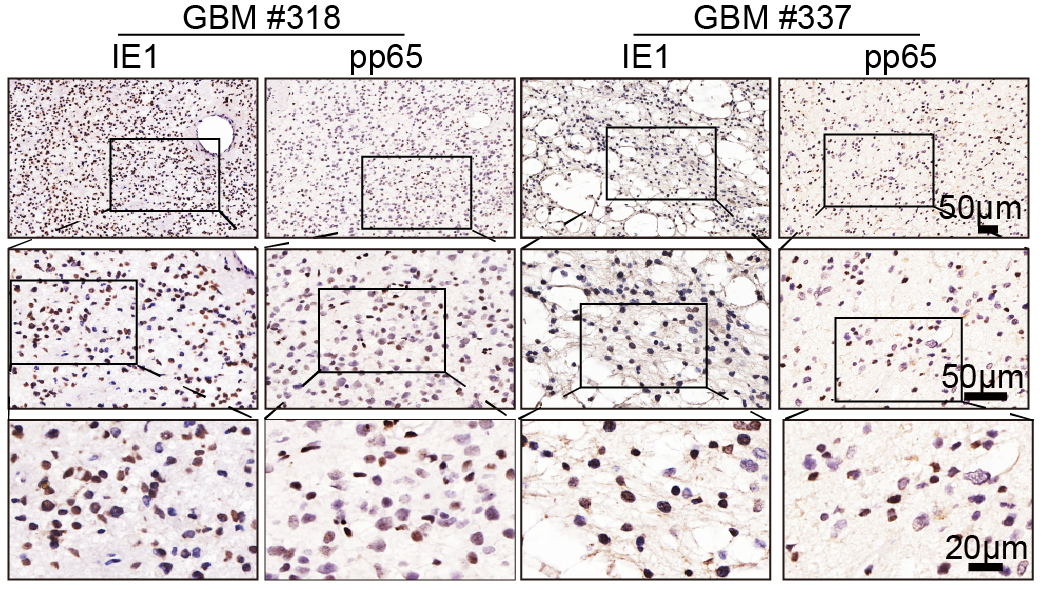

Supplement: S14 Fig — IE1 and pp65 expression in tumor tissues of GBM cases #318 and #337 were tested by IHC. Representative images are shown. (TIF) [file ppat.1011316.s014.tif]

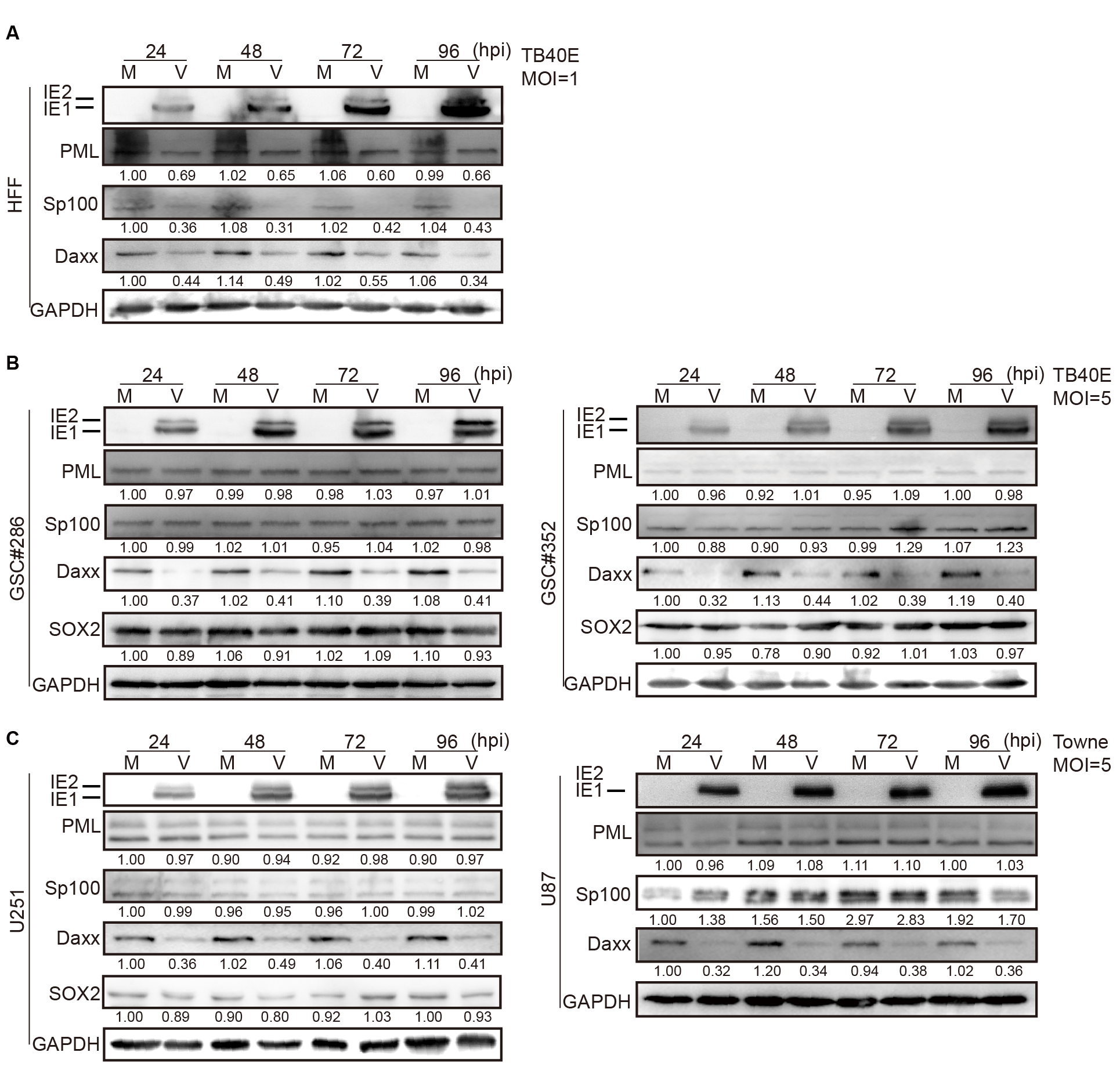

Supplement: S15 Fig — The cells were mock- (M), HCMV Towne strain- or TB40E-infected (V) at an MOI of 1 (for HFFs infection) or 5 (for glioma cell infection) and collected at the indicated times for IB analysis of IE1/2, PML, Sp100, Daxx, and SOX2. HFFs (A), primary glioblastoma cells #286, and #352 (B) were infected with TB40E. U251 and U87 (C) were infected with the Towne strain. GAPDH served as a loading control. The numbers below the blots indicate relative levels of the indicated proteins to those in mock-infected cells at 24 hpi. (TIF) [file ppat.1011316.s015.tif]
