# Supplementary material for: SOX2 downregulation of PML increases HCMV gene expression and growth of glioma cells
Source: PLoS Pathog. 2023 Apr 14;19(4):e1011316. doi: 10.1371/journal.ppat.1011316 (PMC10104302; doi:10.1371/journal.ppat.1011316)
Supplement: S2 Table — (DOCX) [file ppat.1011316.s017.docx]

**S2 Table. Categorical Variable Codings^a^ used in Kaplan-Meier curves shown in Fig 7K**

|  | n | mean | Std. Error | 95% CI from mean |
| --- | --- | --- | --- | --- |
|  |  |  |  |  |
| group IE1_SOX2^b^ | 144 | 30.349 | 2.610 | (15.342-24.658) |
| 1=group: group IE1=0^c^ and SOX2=0 | 32 | 29.020 | 3.498 | (19.192-32.840) |
| 2=group: group IE1=1 and SOX2=0 | 33 | 31.183 | 2.672 | (10.221-55.779) |
| 3=group: group IE1=0 and SOX2=1 | 29 | 39.002 | 5.401 | (10.853-15.147) |
| 4=group: group IE1=1 and SOX2=1 | 50 | 16.708 | 2.087 | (15.342-24.658) |

1. Category variable: group IE1* SOX2 (group IE1_SOX2)
2. Simple Parameter Coding
3. 0：IE1 ^low^; 1：IE1 ^high^;

0：SOX2^low^; 1：SOX2 ^high^
