# Supplementary material for: SOX2 downregulation of PML increases HCMV gene expression and growth of glioma cells
Source: PLoS Pathog. 2023 Apr 14;19(4):e1011316. doi: 10.1371/journal.ppat.1011316 (PMC10104302; doi:10.1371/journal.ppat.1011316)
Supplement: S3 Table — (DOCX) [file ppat.1011316.s018.docx]

Chi-square=36.449, p<0.001

**S3 Table. Variables used in Cox regression analysis to consider association between patient prognosis and IE1/SOX2 levels**

|  | | | | | | 95% CI for Exp (B) | |
| --- | --- | --- | --- | --- | --- | --- | --- |
|  | B | SE | Wald | p | OR | Lower | Upper |
| group_IE1 | 1.635 | .574 | 8.108 | .004 | .195 | 1.665 | 15.813 |
| group_SOX2 | -1.469 | .370 | 15.740 | .000 | .230 | .111 | .475 |
| IE1 * SOX2 |  |  | 11.152 | .004 |  |  |  |
| IE1 * SOX2(1) | 1.762 | .561 | 9.862 | .002 | 5.825 | 1.939 | 17.495 |
| IE1 * SOX2(2) | .293 | .476 | .378 | .000 | 1.340 | .527 | 3.408 |
| IE1 * SOX2(3) | .508 | .661 | .590 | .442 | 1.661 | .455 | 6.608 |
