# Supplementary material for: SOX2 downregulation of PML increases HCMV gene expression and growth of glioma cells
Source: PLoS Pathog. 2023 Apr 14;19(4):e1011316. doi: 10.1371/journal.ppat.1011316 (PMC10104302; doi:10.1371/journal.ppat.1011316)
Supplement: S4 Table — (DOCX) [file ppat.1011316.s019.docx]

Chi-square=28.824, p<0.001

**S4 Table. Assigned coefficients of variables in Cox Regression analysis in Fig 7K**

|  | | | | | | 95% CI | |
| --- | --- | --- | --- | --- | --- | --- | --- |
|  | B | SE | Wald | p | OR | Lower | Upper |
| group IE1 * SOX2 |  |  | 25.599 | **.000** |  |  |  |
| IE1 *SOX2(1) | -.967 | .320 | 9.152 | .002 | .380 | .203 | .711 |
| IE1 *SOX2(2) | -1.465 | .370 | 15.661 | .000 | .231 | .112 | .477 |
| IE1 *SOX2(3) | -1.124 | .330 | 11.626 | .001 | .325 | .170 | .620 |
