# Supplementary material for: SOX2 downregulation of PML increases HCMV gene expression and growth of glioma cells
Source: PLoS Pathog. 2023 Apr 14;19(4):e1011316. doi: 10.1371/journal.ppat.1011316 (PMC10104302; doi:10.1371/journal.ppat.1011316)
Supplement: S5 Table — (DOCX) [file ppat.1011316.s020.docx]

**S5 Table. Characteristics of patients involved in this study**

| Category | Diseases | n | | Gender  (Female/Male) |  | Adults | | Children | | |  |
| --- | --- | --- | --- | --- | --- | --- | --- | --- | --- | --- | --- |
|  |  |  |  |  | n | | Age  Mean (range) | n | n | Age  Mean (range) | |
| HGG | Grade IV (GBM) | 85 | | 30/55 | 62 | | 51 (22-74) |  | 23 | 6 (2-14) | |
|  | Grade III | 109 | | 43/66 | 101 | | 50 (20-79) |  | 8 | 8 (1-15) | |
| LGG | Grade II | 76 | | 39/37 | 71 | | 42 (20-60) |  | 5 | 10 (2-13) | |
|  | Grade I | 8 | | 4/4 | 6 | | 38 (25-48) |  | 2 | 2 (1-3) | |
| NG | Other brain tumor | 162 | | 95/67 | 158 | | 53 (19-78) |  | 4 | 9 (1-3) | |
|  | Non-tumor brain disease | 35 | | 14/21 | 19 | | 36 (18-66) |  | 16 | 7 (1-17) | |
|  | Normal brain tissue | 22 | | 10/12 | 18 | | 55 (20-75) |  | 4 | 5 (1-12) | |
| Totals |  | | 497 | 235/262 | 435 | | 49 (14-79) |  | 62 | 6 (1-13) | |
