# Supplementary material for: SOX2 downregulation of PML increases HCMV gene expression and growth of glioma cells
Source: PLoS Pathog. 2023 Apr 14;19(4):e1011316. doi: 10.1371/journal.ppat.1011316 (PMC10104302; doi:10.1371/journal.ppat.1011316)
Supplement: S6 Table — (DOCX) [file ppat.1011316.s021.docx]

**S6 Table. Antibodies used in this study**

| **Antibodies (clone)** | **Species / Isotype** | **Source / Cat#** |
| --- | --- | --- |
| **Primary antibodies** |  |  |
| Anti-IE1 (clone p63-27) | Mouse monoclonal/IgG_2a_ | Gift from Dr. William J Britt in University of Alabama, U.S. |
| Anti-IE1/2 (clone CH16) | Mouse monoclonal/IgG_1_ | Virusys/P1215 |
| Anti-UL44 (ICP36, CH13) | Mouse monoclonal/IgG_1_ | Virusys/P1202 |
| Anti-pp65 (clone CH12) | Mouse monoclonal/IgG_1_(k) | Virusys/P1205 |
| Anti-pp28 (5C3) | Mouse monoclonal/IgG_2a_ | Virusys/ CA004-1 |
| Anti-SOX2 | Rabbit polyclonal | Abcam/ab97959 |
| Anti-PML | Rabbit polyclonal | proteintech/21041-1-AP |
| Anti-Sp100 | Rabbit polyclonal | proteintech/11377-1-AP |
| Anti-Daxx | Rabbit monoclonal | Abcam/ab32140 |
| Anti-RSAD2 | Rabbit Polyclonal | proteintech/28089-1-AP |
| Anti-IRF9 | Rabbit Polyclonal | proteintech/14167-1-AP |
| Anti-ISG15 | Rabbit Polyclonal | proteintech/15981-1-AP |
| Anti-Ki67 | Rabbit Polyclonal | Abcam/ab15580 |
| Anti-PDGFD | Rabbit polyclonal | proteintech/14075-1-AP |
| Anti-CD31 | Rabbit monoclonal | Abcam/ab222783 |
| Anti-GAPDH | Rabbit polyclonal | proteintech/10494-1-AP |
| **Secondary antibodies** |  |  |
| Alexa Fluor 594 conjugate Goat anti-Mouse IgG1 | Goat | Invitrogen/A-21125 |
| Alexa Fluor 647-conjugated goat anti-rabbit IgG (H+L) | Goat | Invitrogen/A-21244 |
| Alexa Fluor 488-conjugated goat anti-rabbit IgG (H+L) | Goat | Invitrogen/A11008 |
| Alexa Fluor 488-conjugated goat anti-mouse IgG1 | Goat | Invitrogen/A-21121 |
| Peroxidase-anti-Mouse IgG | Goat | Jackson ImmunoResearch  Laboratories/115-035-003 |
| Peroxidase-anti-Rabbit IgG | Goat | Jackson ImmunoResearch Laboratories/111-035-003 |
