# Supplementary material for: SOX2 downregulation of PML increases HCMV gene expression and growth of glioma cells
Source: PLoS Pathog. 2023 Apr 14;19(4):e1011316. doi: 10.1371/journal.ppat.1011316 (PMC10104302; doi:10.1371/journal.ppat.1011316)
Supplement: S7 Table — (DOCX) [file ppat.1011316.s022.docx]

**S7 Table. sgRNA sequences used in this study**

|  | **Target name** | **Sequences (5’-3’)** |
| --- | --- | --- |
| SOX2 | SOX2 gRNA#1 | TCGCCATGCTATTGCCGCCG |
|  | SOX2 gRNA#2 | GCTATTGCCGCCGGGTTTTAG |
| PML | PML gRNA#1 | GTGAACCGCGCCAAGTTTTA |
|  | PML gRNA#2 | CAGCGCGACTACGGTTTTAG |
| Sp100 | Sp100 gRNA#1 | GATGAGATCACGATCACGGG |
|  | Sp100 gRNA#2 | CACGATCACGGAGGCCCTCG |
