# Supplementary material for: SOX2 downregulation of PML increases HCMV gene expression and growth of glioma cells
Source: PLoS Pathog. 2023 Apr 14;19(4):e1011316. doi: 10.1371/journal.ppat.1011316 (PMC10104302; doi:10.1371/journal.ppat.1011316)
Supplement: S8 Table — (DOCX) [file ppat.1011316.s023.docx]

**S8 Table. qPCR Primers used in this study**

| Gene | Primer | Sequence (5’-3’) | Product size (bp) |
| --- | --- | --- | --- |
| SOX2 [1] | F | GCCGAGTGGAAACTTTTGTCG | 154 |
|  | R | GCAGCGTGTACTTATCCTTCTT |  |
| PML | F | AGCCCTACAGAGCGAGCCCCCG | 191 |
|  | R | GCGGGTGTGTCTGCACCTAG |  |
| Sp100 | F | TGAGCACCAGGAGGCTGAAT | 259 |
|  | R | TCTTGAGAATCTTCAAACAT |  |
| ISG15 | F | TCCAGCAGCGTCTGGCTGTC | 190 |
|  | R | ACGGTCTGCGTCAGCCGTAC |  |
| RSAD2 | F | GAATTATGGTGAGTATTTGGAC | 185 |
|  | R | CGTTGAAACGATTAATGACAG |  |
| IFR9 | F | CCTTCTTCAAGGCCTGGGCA | 205 |
|  | R | CCTGGCTGGCCAGAGACGAT |  |
| GAPDH [1] | F | GAGTCAACGGATTTGGTCGT | 185 |
|  | R | GACAAGCTTCCCGTTCTCAG |  |
| UL123 [2] | F | GCCTTCCCTAAGACCACCAAT | 101 |
|  | R | ATTTTCTGGGCATAAGCCATAATC |  |
| UL44 [2] | F | GTCGGGAACAGCGGCAAGTC | 154 |
|  | R | CGCCACCTCCTCCCAGACC |  |
| UL99 [2] | F | GGGTCGCCAGGTGTCTCTACG | 166 |
|  | R | GCTGCCGCTACTATTGTCGTTTCC |  |

**Reference**

1 Wu CC, Jiang X, Wang XZ, Liu XJ, Li XJ, Yang B *et al*. Human Cytomegalovirus Immediate Early 1 Protein Causes Loss of SOX2 from Neural Progenitor Cells by Trapping Unphosphorylated STAT3 in the Nucleus. *J Virol* 2018; 92.

2 Cheng S, Jiang X, Yang B, Wen L, Zhao F, Zeng WB *et al*. Infected T98G glioblastoma cells support human cytomegalovirus reactivation from latency. *Virology* 2017; 510: 205-215.
